# The effects of traditional Chinese botanical medicine on membranous nephropathy

**DOI:** 10.3389/fphar.2026.1797220

**Published:** 2026-04-13

**Authors:** Tong Yin, Xinyu Zhang, Jiahao Liang, Xiaoming Yan, Zepeng Li, Wenlong Li, Jingyi Li, Lianhua Li, Ming Chen

**Affiliations:** 1 Heilongjiang Academy of Chinese Medicine Sciences, Harbin, China; 2 Graduate School, Tianjin University of Traditional Chinese Medicine, Tianjin, China; 3 Graduate School, Heilongjiang University of Chinese Medicine, Harbin, China

**Keywords:** Chinese botanical medicines, clinical efficacy, mechanism, membranous nephropathy, review, signal pathway

## Abstract

Membranous nephropathy (MN), an autoimmune glomerulopathy driven by podocyte antigens, is a leading cause of end-stage renal disease (ESRD). However, its pathogenic mechanisms remain incompletely understood. This knowledge gap results in significant physical and psychological burdens for patients worldwide and poses substantial challenges for clinical management. Current conventional treatments for MN are often associated with significant side effects and may not yield satisfactory outcomes. Therefore, the development of more efficacious and better-tolerated therapeutic strategies for MN is imperative. Given these clinical challenges, Traditional Chinese Botanical Medicine (CHMs), with their multi-component and multi-target characteristics, have emerged as a promising alternative or complementary therapeutic approach for MN. This review aims to summarize the evidence on the mechanisms and clinical efficacy of CHMs and their active metabolites in treating MN, drawing from findings reported in animal experiments, clinical trials, systematic reviews, and meta-analyses. It seeks to elucidate the potential advantages of CHMs in MN management and to provide a reference for future research in this field. Study design and methods: For this review, the following major academic research databases were consulted: PubMed, ResearchGate, Science Direct, and Web of Science. “Glomerulonephritis, Membranous [MeSH Terms] OR Extramembranous Glomerulopathy [Text Word] OR Membranous Glomerulonephropathy [Text word] OR Membranous Nephropathy [Text word] OR Idiopathic Membranous Glomerulonephritis [Text word]OR Glomerulonephritides, Idiopathic Membranous. [Text word]),” “(Medicine, Chinese Traditional [MeSH Terms] OR Medicine, Chinese Traditional [Text Word] OR Medicine, Chinese Traditional [Text word] OR Tongue Diagnosis, Traditional [Text word] OR Traditional Tongue Assessment [Text word],” “Drugs, Chinese Herbal [MeSH Terms] OR Chinese Drugs, Plant [Text Word] OR Chinese Herbal Drugs [Text word] OR Plant Extracts, Chinese [Text word] OR Chinese Plant Extracts [Text word],” “mechanism,” “Meta-analysis,” “systematic review,” “RCT,” “botanical drug” and their combinations were the keywords to search the relevant literature. Data were collected from 2019 to 2025.

## Graphical Abstract

**Figure:**
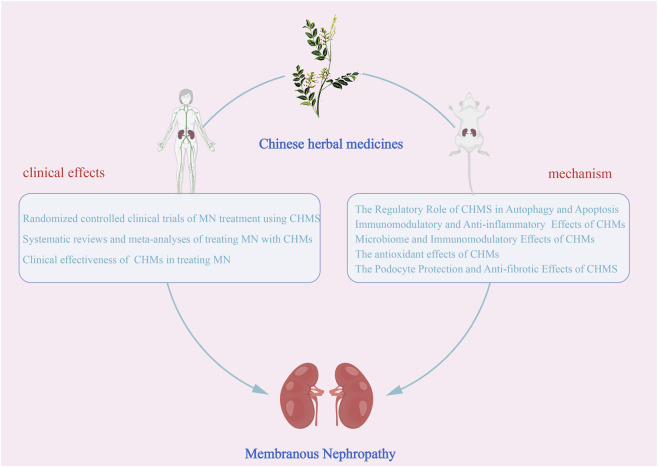

## 1 Introduction

MN, a primary glomerular disease, is responsible for about 30% of adult nephrotic syndrome cases. In these cases, around 80% of patients lack a definitive etiology (Ronco et al., 2021). Additionally, MN exhibits a peak incidence within the middle-aged and elderly demographic, and its prevalence continues to rise amid the global aging trend (Qin et al., 2023). In Central China, the incidence of MN increased from 15.98% in 2009 to 30.81% in 2018, surpassing IgA nephropathy to emerge as the leading pathological category of primary glomerulopathy in the adult Chinese demographic (Hu et al., 2020), While approximately 30% of those diagnosed attain spontaneous disease remission, a matching proportion goes on to develop end-stage renal disease (ESKD) (Alsharhan and Beck, 2021).

**FIGURE 1:**
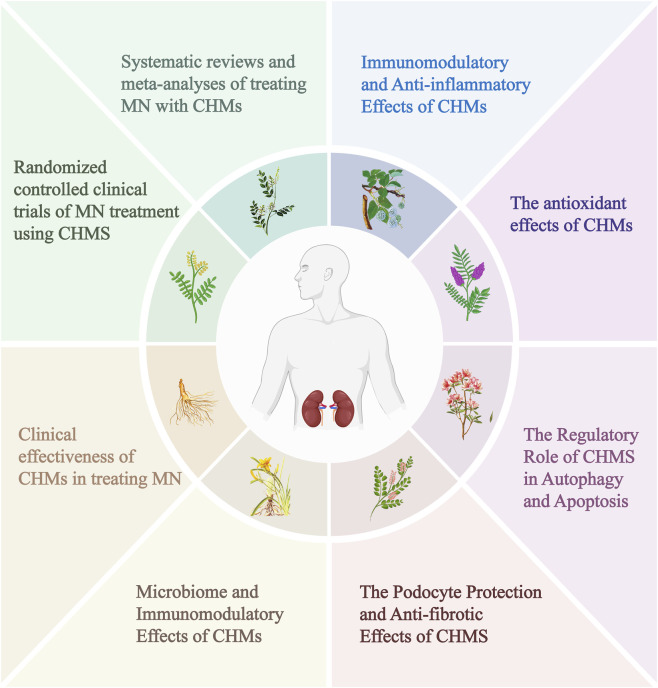
Overview.

MN is an autoimmune glomerular diseasewith 70%–80% of cases associated with the PLA2R antigen, 3%–5% linked to the THSD7A antigen, and the remaining antigens yet to be identified. A hallmark histopathological trait of MN is the accumulation of immune complexes underneath the glomerular epithelial layer, inducing glomerular basement membrane thickening alongside the classic “spiked” morphological alterations (Beyze et al., 2024; Zhang et al., 2024d). Clinically, MN is often characterized primarily by nephrotic syndrome, with some patients presenting concurrent microscopic hematuria (Ronco et al., 2021; Ragy et al., 2025). It is noteworthy that as high as 49% of newly diagnosed cases of MN already have proteinuria (Hamilton et al., 2023), and the onset of proteinuria can precede clinical diagnosis by several years, which confirms the existence of a subclinical phase of the disease. However, research into the pathogenesis of MN remains in its early stages, which directly contributes to the current diagnostic bottleneck. Currently, a stepwise treatment strategy is adopted for patients with MN, with the core principle of providing targeted treatment based on the patient’s risk of disease progression (Xu et al., 2025). Patients at low risk are treated with supportive care, such as the use of RAS inhibitors (ACEI/ARB) (Bharati et al., 2024). Although such agents can reduce proteinuria, their use is linked to a heightened risk of hyperkalemia. Additionally, they may precipitate an acute decline in renal function (Villa-Zapata et al., 2021). Among patients with a moderate-to-high-risk profile, rituximab is the first-line treatment, achieving a remission rate of 64%–74%, though 20%–25% of patients experience relapse. Cyclophosphamide is the second-line option, but its carcinogenic risk increases with cumulative dose (Seifert et al., 2024; Fervenza et al., 2019). Calcineurin inhibitors such as tacrolimus and cyclosporine, while effective for patients at moderate to high risk, exhibit recurrence rates of 30%–40% and are generally not the primary immunosuppressive choice for this patient group (Canney et al., 2025). Thus, the scarcity of effective therapeutic options and the adverse effects of current medications lead to an unfavorable prognosis in MN patients, who are prone to disease progression (Matsuzaki et al., 2023). MN may eventually progress to ESKD (Hullekes et al., 2024). Studies have reported that the 4-year mortality rate of ESKD patients is as high as 26.5% (Shi et al., 2025). Given the side effects of conventional immunosuppressive therapies, safer and more effective treatments for MN are urgently needed. Natural products such as propolis and pollen have been shown to confer renoprotection in hypertensive models through antioxidant and anti-inflammatory activities (Salmas et al., 2017; Talas et al., 2014). Similarly, synthetic organoselenium compounds exhibit protective effects against renal oxidative stress (Talas et al., 2009). These findings highlight the therapeutic potential of natural products, laying a foundation for exploring CHMs in the management of MN. In recent years, CHMs have emerged as a promising and evidence-based therapeutic strategy for MN, capable of significantly alleviating proteinuria and preserving renal function (Shan et al., 2024). CHMs contain diverse bioactive metabolites and exert their effects via multi-target synergistic regulation *in vivo*. Therefore, both early diagnosis and effective clinical therapies for MN urgently require comprehensive summarization, and further in-depth and systematic investigations into its pathogenic mechanisms are warranted. Meanwhile, the compatibility of TCM botanical drugs enables individualized treatment. These properties expand the potential applications of TCM in MN therapy. Accordingly, this review synthesizes current understanding of how Chinese medicine monomers, metabolites, and formulas exert their effects in MN treatment, especially in terms of antioxidation, anti-inflammation, anti-fibrosis, immunomodulation, autophagy, and regulation of cell apoptosis. Furthermore, this article reviews recent clinical evidence on the efficacy of Chinese medicine for MN (Figure 1).

## 2 Mechanism of CHMs in the treatment of MN

### 2.1 Immunomodulatory and anti-inflammatory effects of CHMs

Excessive inflammatory activation and immune dysfunction constitute the core pathological basis for the onset and progression of MN (Pang et al., 2025). For example, podocyte antigen-mediated autoimmune reactions trigger immune complex deposition, which in turn activates the complement system, inflammatory signaling pathways, and dysregulation of immune cell subsets.

#### 2.1.1 NF-κB signaling pathway

Recent studies have shown that NF-κB signaling serves as a core signaling hub in the pathological injury of MN by promoting the activation of the complement system, podocyte antigen expression, and local renal inflammation, such as glomerular inflammation. Its abnormal activation is a key link driving podocyte injury and systemic immune dysregulation (Xie et al., 2020).

Zhen-Wu-Tang Decoction (ZWT) is a classic formula in TCM, which first appeared in the Treatise on Febrile Diseases. It is composed of *Aconitum carmichaelii* Debeaux [Ranunculaceae; Aconiti lateralis radix praeparata], *Poria cocos* (Schw.) Wolf [Polyporaceae; Poria], *Atractylodes macrocephala* Koidz [Asteraceae; Atractylodis macrocephalae rhizoma], *Paeonia lactiflora* Pall [Paeoniaceae; Paeoniae radix alba] and *Zingiber officinale* Roscoe [Zingiberaceae; Zingiberis rhizoma recens]. In recent years, a growing body of experimental evidence has confirmed that ZWT can inhibit the activation of the NF-κB signaling pathway (Li et al., 2020; Fang et al., 2024). In 2019, Liu et al. treated TNF-α-induced damaged podocytes with serum containing ZWT and found that ZWT could reduce p-p65, phosphorylation of p-IκBα, and expression of NLRP3, Caspase-1, and IL-1β, inhibit the colocalization of NLRP3 with apoptosis-associated speck-like protein (ASC), and block activation of the NF-κB/NLRP3 signaling axis, thereby exerting nephroprotective effects. Furthermore, in C-BSA-induced MN rats treated with ZWT via oral administration, it was observed that ZWT (low, medium, and high doses of 4.2 g/kg,8.4 g/kg, 16.8 g/kg for 4 weeks) reduced 24-h urine protein excretion (24h-UPE), serum total cholesterol (TC), and triglycerides (TG). It also exerted a mitigating effect on glomerular basement membrane (GBM) thickening, decreased IgG and C3 deposition, and mitigated inflammatory responses and podocyte injury in the MN (Liu et al., 2019).

As a bioactive substance of natural derivation, Diosgenin (DG) is obtained from Chinese yam (Rhizoma dioscoreae), a medicinal botanical drug widely utilized in TCM practice (Zhong et al., 2023), extensively utilized for the therapeutic management of diabetes and nephropathy (Zhong et al., 2022; Wang et al., 2022c). To investigate whether DG exerts therapeutic effects on MN and its specific mechanisms, Jia et al. administered DG (administered orally by gavage at a dose of 10 mg/kg per day for 4 weeks) to C-BSA-induced MN rats in 2024. Their experimental findings demonstrated that DG suppressed the nuclear translocation of NF-κB p65 and downregulated the expression of pro-inflammatory proteins, including IKKβ and ICAM-1, while triggering the Nrf2/Keap1 signaling pathway and promoting the expression of NQO1 and HO-1, thereby exerting protective effects on MN kidneys. Besides, DG diminished oxidative stress in the kidneys by increasing SOD and GSH activities and decreasing MDA levels, while also ameliorating renal function in MN rats through reducing urinary protein, SCr, and BUN levels (Jia et al., 2024).

Sanqi Oral Solution (SQ) is a commonly used TCM formulation clinically applied in China for treating chronic kidney disease (Hu et al., 2024a). It is composed of *Astragalus membranaceus* (Fisch.) Bge. [Fabaceae; Astragali radix] and *Panax notoginseng* (Burk.) F.H. Chen [Araliaceae; Notoginseng radix et rhizoma]. In 2019, Tian et al. discovered in a C-BSA-induced MN rat model that SQ (administered via gavage at 6.3 mL/kg/d for 4 weeks) could suppress NF-κB signaling pathways, downregulating NF-κB p65 and its phosphorylated forms, suppressing inflammatory responses, reducing proteinuria excretion, elevating ALB levels, mitigating injuries such as glomerular enlargement and capillary wall thickening, decreasing glomerular C3 and IgG deposition, restoring podocin and synaptopodin expression, alleviating podocyte foot process fusion and GBM thickening, thereby attenuating glomerular damage in MN rats (Tian et al., 2019).

Modified Huangqi Chifeng Decoction (MHCD) is a TCM formula composed of *A. membranaceus* (Fisch.) Bge [Fabaceae; Astragali radix], *Euryale ferox* Salisb [Nymphaeaceae; Euryales semen], *Rosa laevigata* Michx [Rosaceae; Rosae laevigatae fructus], *P. lactiflora* Pall [Paeoniaceae; Paeoniae radix rubra], *Saposhnikovia divaricata* (Turcz. ex Ledeb.) Schischk [Apiaceae; Saposhnikoviae radix], *Hedyotis diffusa* Willd. [Rubiaceae; Hedyotis diffusae herba] e *Dioscorea nipponica* Makino [Dioscoreaceae; Dioscoreae nipponicae rhizoma]. In 2025, Chang et al. found in a PHN rat model induced by sheep anti-rat Fx1A serum that MHCD(12.5 g/kg/d administered by gavage for 6 weeks) is capable of downregulating the activation process of the NF-κB pathway, suppressing the protein expression of phosphorylated NF-κB p65, enhancing the expression levels of nephrin, podocin, and WT-1, and diminishing the deposition of IgG as well as membrane attack complex C5b-9, decreasing urinary protein and TG levels, increasing ALB content, and alleviating glomerular pathological damage (Chang et al., 2025).

Apart from exerting a direct downregulatory effect on the NF-κB pathway, some CHMs can also exert their effects by regulating its upstream signaling molecules. Aryl hydrocarbon receptor (AHR), serving as a sensing molecule for environmental toxins and intrinsic metabolites (Panda et al., 2023), can aggravate inflammatory reactions in renal tissues via the NF-κB pathway upon abnormal activation (Barroso et al., 2021; Wang et al., 2023d). In 2023, Ma et al. found in a C-BSA-induced MN rat model that MSG (3.70 g/kg intragastric administration for 4 weeks) could regulate the NF-κB/Nrf2 signaling pathways by inhibiting AHR signaling, downregulate the expression of AHR downstream target genes CYP1A1, CYP1A2, CYP1B1, COX-2 and NF-κB downstream inflammatory factors MCP-1, iNOS, upregulate the expression of Nrf2 downstream antioxidant genes HO-1, CAT, reduce the infiltration of F4/80+ and CD3+T cells in renal tissues, restore the expression of podocin, nephrin, synaptopodin, thereby inhibiting inflammatory responses and oxidative stress, alleviating podocyte injury, and improving proteinuria (Ma et al., 2023).

Astragaloside IV (AS-IV) is a naturally occurring phytochemical derived from *A. membranaceus* (Fisch.) Bge. [Fabaceae; Astragali radix]—a botanical drug widely employed in TCM. This bioactive agent possesses anti-inflammatory and antioxidant properties, while also regulating cellular autophagy and apoptotic processes (Chen et al., 2025; Zhang et al., 2024a; Yang et al., 2025). To clarify the potential molecular mechanism of AS-IV in MN, Ma et al. performed intragastric administration of AS-IV (20, 40 mg/kg) for 4 weeks in Sprague-Dawley rats with passive Heymann nephritis (PHN). The findings indicated that AS-IV reduced levels of BUN, SCr, and 24h-UPE, while increasing ALB concentration. Additionally, AS-IV alleviated IgG deposition, attenuated pathological injuries, including GBM thickening, podocyte foot process effacement, and renal interstitial fibrosis, and diminished the expression of TNF-α, IL-6, and IL-1β. Further *in vitro* experiments using a TNF-α-induced MPC5 podocyte model revealed that AS-IV promoted K48-linked ubiquitination and degradation of TRAF6, inhibited phosphorylation and nuclear translocation of NF-κB p65, downregulated the expression of TNF-α, IL-6, and IL-1β, significantly enhanced podocyte viability, and mitigated the inflammatory response (Ma et al., 2024).

**FIGURE 2:**
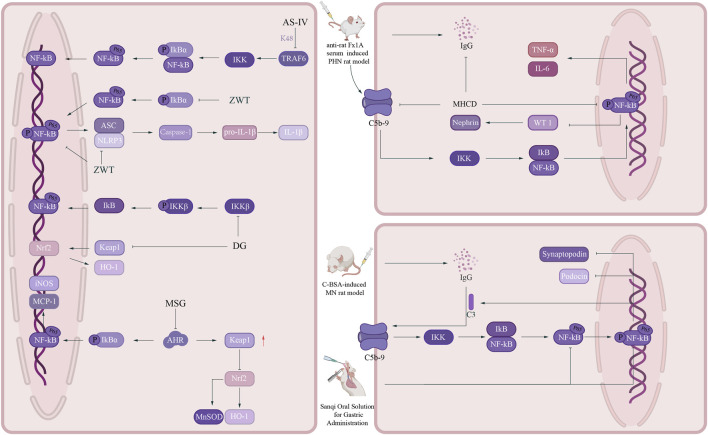
Schematic diagram of CHMs modulating the NF-κB signaling pathway NF-κB: nuclear factor kappa-B. IκB: inhibitor of NF-κB IκBα:inhibitor of κB α TRAF6:tumor necrosis factor receptor-associated factor 6 NLRP3:NOD-like receptor pyrin domain-containing protein 3 ASC:apoptosis-associated speck-like protein containing a CARD IKKβ:inhibitor of κB kinase β IL-1β:Interleukin-1β Keap1:Kelch-like ECH-associated protein 1 HO-1:heme oxygenase-1 AHR: aryl hydrocarbon receptor Nrf2:nuclear factor erythroid 2-related factor 2 MnSOD: manganese superoxide dismutase TNF-α: tumour necrosis factor α WT-1:Wilms tumor 1

Safranal, an active metabolite of *Crocus sativus* L. [Iridaceae; Croci stigma] (Ali et al., 2024), is a natural monoterpenal metabolite (Yan et al., 2024a)with significant anti-inflammatory and antioxidant activities (Bahari et al., 2025; Abdian et al., 2024). In 2025, Bao et al. found in a C-BSA-induced rat model of MN that Safranal (low and high doses of 100, 200 mg/kg/d via intragastric administration for 4 weeks) activates SIRT1 to mediate NF-κB p65 deacetylation, in turn suppressing the NF-κB signaling pathway. This is accompanied by reduced phosphorylated protein expression of p-p65 and p-IκBα, diminished secretion of IL-6 and TNF-α, enhanced expression levels of podocyte-specific marker proteins (podocin, nephrin, and WT-1), and mitigated deposition of IgG and C3—ultimately alleviating inflammatory responses, protecting podocyte integrity, and attenuating renal injury in MN rats (Bao et al., 2025) (Figure 2).

#### 2.1.2 JAK-STAT signaling pathway

Dysregulated activation of the JAK-STAT pathway markedly accelerates MN progression. Critical cytokines—including IL-6, a key mediator in renal inflammation—engage with cell-specific receptors on the surface of kidney parenchymal cells, inducing gp130 homodimerization, activating JAK kinases and their downstream STAT phosphorylation cascade, thereby inducing multiple downstream effects. On one hand, the transcriptional upregulation of IL-6, NF-α, L-4, and TGF-β1 exacerbates local renal inflammation, immune complex deposition, and fibrosis. On the other hand, sustained activation of this pathway—particularly through excessive JAK2 activation—Results in abnormalities in the expression of nephrin and other key functional proteins, coupled with compromised autophagic activity. This further intensifies podocyte injury, proteinuria, and drives overall disease progression (Long et al., 2025; Yuan et al., 2022).

Dysregulated immune reactions and podocyte damage represent two core pathogenic drivers underlying the development of MN, while the IL-6/STAT3 signaling pathway serves as a pivotal regulator in both of these pathogenic processes. Zhao et al. found that treatment with Ma Huang Fu Zi He Shen Zhuo Tang (MFSD) significantly reduced IL-6 levels in MN patients. In a PHN rat model, MFSD (1 mL/100 g/d administered via gavage for 12 weeks) was shown to inhibit transcription of key genes in the IL-6/STAT3 pathway and STAT3 phosphorylation, upregulate Bcl-2, and decrease IL-4 and IFN-γ levels, restored podocin, nephrin, and synaptopodin expression, reduced proteinuria and dyslipidemia, and improved rena (Zhao et al., 2024). Compared to the limited efficacy of the single-target biologic tocilizumab in blocking the IL-6R signaling pathway in PHN rats (Zhao et al., 2024), MFSD demonstrates the unique advantage of TCM formulas in integrating and regulating complex signaling networks by synergistically inhibiting key nodes of the IL-6/STAT3 pathway through multi-targeted action.

Shengyang Yiwai Decoction is a classic formula comprising 16 botanical drugs, including *A. membranaceus* (Fisch.) Bge. [Fabaceae; Astragali radix], *Panax ginseng* C.A.Mey. [Araliaceae; Ginseng Radix], *A. macrocephala* Koidz [Asteraceae; Atractylodis Macrocephalae Rhizoma], *Poria cocos* (Schw.) Wolf [Polyporaceae; Poria], *P. lactiflora* Pall. [Paeoniaceae; Paeoniae Radix Alba], *Pinellia ternata* (Thunb.) Breit [Araceae; Pinelliae Rhizoma], *Alisma orientale* (Sam.) Juz [Alismataceae; Alismatis Rhizoma], *Glycyrrhiza uralensis* Fisch. [Fabaceae; Glycyrrhizae Radix et Rhizoma Praeparata], *Citrus reticulata* Blanco [Rutaceae; Citri Reticulatae Pericarpium], *Notopterygium incisum* Ting ex H.T.Chang [Apiaceae; Notopterygii Rhizoma et Radix], *Angelica pubescens* Maxim. [Apiaceae; Angelicae Pubescentis Radix], *S. divaricata* (Turcz.) Schischk. [Apiaceae; Saposhnikoviae Radix], *Bupleurum chinense* DC. [Apiaceae; Bupleuri Radix], *Coptis chinensis* Franch. [Ranunculaceae; Coptidis Rhizoma], *Z. officinale* Roscoe [Zingiberaceae; Zingiberis Rhizoma Recens] and *Ziziphus jujuba* Mill [Rhamnaceae; Jujubae Fructus]. It originates from Li Dongyuan’s Treatise on the Spleen and Stomach during the Jin-Yuan period. In 2025, Wu et al. demonstrated in a PHN rat model that Shengyang Yigwei Decoction (12 g/kg/d administered via gastric lavage for 6 weeks)downregulated IL-4 and GATA3 levels in renal tissue while upregulating IFN-γ and T-bet expression, corrected Th1/Th2 immune imbalance, and reducing p-JAK2/JAK2 and p-STAT3/STAT3 phosphorylation, inhibiting JAK2/STAT3 signaling pathway activation. It also increased synaptopodin, nephrin, and podocalyxin expression, consequently reducing 24h-UPE, decreasing glomerular IgG deposition, and improving GBM thickening and inflammatory cell infiltration (Wu et al., 2025).

Colquhounia Root Tablet (CRT) is a clinically approved prescription prepared from the peeled roots of *Tripterygium hypoglaucum* (Levl.) Hutch [Celastraceae; Tripterygii hypoglaucae radix] (Ma et al., 2022). It exhibits significant anti-inflammatory and immunomodulatory biological activities (Li et al., 2022a; Schcolnik-Cabrera et al., 2019) and is commonly utilized for the therapeutic intervention of glomerular diseases in China (Xu et al., 2024b). In 2023, Mao et al. demonstrated in C-BSA-induced MN mouse models and C5b-9-induced MPC-5 injury models that CRT (*in vitro*: 186/372/744 μg/mL) downregulated TNF-α, IL-6, and MMP9 expression, reduced JAK2 and STAT3 phosphorylation levels, and upregulated nephrin and podocin expression, reduce glomerular basement membrane thickening, IgG deposition, and mesangial cell proliferation, thereby mitigating renal injury and immune inflammatory responses and improving MN symptoms (Mao et al., 2023).

#### 2.1.3 Complement and humoral immunity

Dysregulated activation of the complement cascade constitutes a core pathogenic mechanism driving the progression of MN, which is intimately linked to podocyte damage with structural impairment, impairment of glomerular filtration barrier integrity, and an unfavorable prognosis (Duan et al., 2025). Additionally, abnormal complement activation is associated with humoral immunity. B cells secrete anti-PLA2R1/THSD7A IgG antibodies, which bind to glomerular antigens to form immune complexes. These complexes trigger the complement cascade, ultimately generating anaphylatoxins C3a, C5a, and C5b-9 (Kistler and Salant, 2024; Seifert et al., 2023). These complement activation products exacerbate pathological damage through a dual mechanism: on one hand, C3a and C5a induce inflammatory injury by activating corresponding receptors on podocytes; on the other hand, C5b-9 directly inserts into podocyte membrane structures, compromising the architectural integrity of the glomerular filtration barrier (Chen et al., 2023a). Clinical analysis further validated the pathological significance of complement activation. In MN patients, C5b-9 deposition in glomeruli showed a significant positive correlation with proteinuria (r = 0.88, P < 0.0001) and showed an inverse correlation with eGFR (Avasare et al., 2024; Sethi et al., 2023).

Total coumarin derivatives from Hydrangea paniculata (HP) are bioactive metabolites derived from the classic Chinese medicinal botanical drug Hydrangea paniculata, exerting potent anti-inflammatory and antifibrotic properties (Wang et al., 2022a). In 2022, Wang et al. established a C-BSA-induced rat MN model and administered different doses of HP (low, medium, and high doses of 7.5, 15, and 30 mg/kg via oral gavage for 9 weeks). They found that HP suppressed complement activation, reduced C3 autocrine secretion and autoclyolysis in renal tubular cells, and decreased C3b deposition. Concurrently, HP downregulated IL-10 secretion by impeding the PI3K-AKT and NF-κB signaling transduction pathways, reduced CD20 expression, thereby suppressing IL-10-mediated interstitial fibrosis and mitigating humoral immune injury (Wang et al., 2022a). Furthermore, in a 28-day toxicity study, rats treated with HP (450 mg/kg) showed no significant adverse reactions.

Polyamide resin purified corn silk ethanol extract (PR-CSEE) is an ethanol extract of corn silk flavonoids (CSFs), the active metabolites of the TCM Stigma maydis. Wang et al. found in a C-BSA-induced murine model of MN that PR-CSEE (administered via oral gavage at a dose of 20 mg TFC/kg for 4 weeks) significantly reduced 24 h-UPE, Scr, and TG levels, elevated ALB and BUN levels by decreasing the levels of IgG and C3, alleviating GBM thickening and inflammatory cell infiltration, thereby exerting a therapeutic effect on MN (Wang et al., 2021b).

Yishen’an (YSA) is a compound formula composed of *P. notoginseng* (Burkill) F.H. Chen [Araliaceae; Notoginseng Radix et Rhizoma], *Rheum officinale* Baill [Polygonaceae; Rhei Radix et Rhizoma], *Salvia miltiorrhiza* Bunge [Lamiaceae; Salviae Miltiorrhizae Radix et Rhizoma], *Lonicera confusa* (Sweet) DC. [Caprifoliaceae; Lonicerae Japonicae Flos], *Carthamus tinctorius* L. [Compositae; Carthami Flos], *Forsythia suspensa* (Thunb.) Vahl [Oleaceae; Forsythiae Fructus], *Scutellaria barbata* D. Don [Labiatae; Scutellariae Barbatae Herba], *Wolfiporia extensa* (Peck) Ginns [Polyporaceae; Poria], and *G. uralensis* Fisch. [Leguminosae; Glycyrrhizae Radix et Rhizoma]. Zhao et al. found in a C-BSA-induced MN rat model that YSA (at low, medium-low, medium-high, and high doses of 0.25 g/kg, 0.5 g/kg, 1 g/kg, 2 g/kg for 35 days) reduced the generation of active fragments such as C3c, lowered CIC levels, restored the decreased IgG levels in the model group while reducing IgM levels, inhibited immune complex deposition, significantly decreased 24h-UPE, increased SOD activity, reduced MDA levels, improved oxidative stress status, and regulated lipid indicators including TC and TG (Zhao et al., 2022). This experiment demonstrates that YSA exerts an anti-MN effect by inhibiting the humoral immune response and complement activation.

#### 2.1.4 Immune cell regulation

The pathogenesis of MN is closely associated with T cell subset dysregulation (Li et al., 2023c). Its core features include immune cell infiltration and tissue damage caused by abnormal activation of Th17 cells, as well as disrupted immune tolerance due to Treg functional suppression, encompassing autoantibody production and immune complex deposition (Zhao et al., 2021).

QingreHuoxue Formula consists of *Codonopsis pilosula* (Franch.) Nannf [Campanulaceae; Codonopsis Radix], *A. macrocephala* Koidz. [Asteraceae; Atractylodis macrocephalae Rhizoma], *S. miltiorrhiza* Bunge [Lamiaceae; Salviae miltiorrhizae Radix et Rhizoma], *H. diffusa* Willd. [Rubiaceae; Hedyotidis Herba], *Scutellaria baicalensis* Georgi [Lamiaceae; Scutellariae Radix], *Pyrrosia lingua* (Thunb.) Farw [Polypodiaceae; Pyrrosiae Folium], *Plantago asiatica* L [Plantaginaceae; Plantaginis Herba], *Polyporus umbellatus* (Pers.) Fr. [Polyporaceae; Polyporus], *Angelica sinensis* (Oliv.) Diels [Apiaceae; Angelicae sinensis Radix], *Leonurus japonicus* Houtt [Lamiaceae; Leonuri Herba] and *Astragalus mongholicus* Bunge [Fabaceae; Astragali Radix]. In 2024, Lou et al. demonstrated in a C-BSA-induced MN mouse model that the Qingre Huoxue Formula (administered orally at 19.44 g/kg for 4 weeks) could reduce proteinuria and IgG expression by modulating peripheral blood Th17 cell and Treg populations, thereby downregulating IL-17 and TGF-β1 (Lou et al., 2024). Additionally, the study found that the botanical formula for clearing heat and promoting blood circulation demonstrated comparable efficacy to Western medications. However, when combined with prednisolone acetate and cyclosporine A, it yielded superior results compared to Western medication alone, while also reducing the side effects and drug dependency associated with immunosuppressants.

### 2.2 The antioxidant effects of CHMs

Renal oxidative stress serves a pivotal pathogenic function in the initiation and progression of MN (Kishi et al., 2024; Nezu and Suzuki, 2020). When the kidneys in MN are stimulated by factors such as ischemia-reperfusion injury and high glucose, triggering renal vascular abnormalities, it leads to mitochondrial respiratory chain dysfunction, thereby resulting in excessive production of ROS. Excessive ROS disrupts the oxidative-antioxidative balance and directly damages glomerular podocytes, endothelial cells, and the basement membrane (Zhu et al., 2022; Ho and Shirakawa, 2022). Excessive oxidative stress can disrupt the Keap1-Nrf2 system in MN, while SIRT1 enhances antioxidant defense by activating Nrf2, thereby mitigating foot cell damage and delaying MN progression (Perico et al., 2024).

The critical role of oxidative stress in MN pathogenesis highlights the potential value of exogenous antioxidants as therapeutic agents. Indeed, natural products with antioxidant properties have demonstrated protective effects against renal oxidative injury in various experimental models. For instance, propolis and its active metabolite caffeic acid phenethyl ester (CAPE) were shown to ameliorate kidney damage in hypertensive rats by improving total antioxidant status and reducing oxidative stress markers (Salmas et al., 2017). Additionally, propolis has been reported to attenuate oxidative injury in both kidney and heart tissues induced by nitric oxide synthase inhibition (Talas et al., 2014). These findings support the rationale for investigating antioxidant-rich CHMs in the treatment of MN, where oxidative damage is a key driver of podocyte injury and disease progression.

Curcumin is a natural phytochemical isolated from turmeric root (Liu et al., 2023a) that exhibits significant antioxidant activity (Sadeghi et al., 2023; Hu et al., 2023). In 2020, Tu et al. found in the PHN rat model induced by anti-Fx1A serum that curcumin (300 mg/kg/d by gavage for 30 days) could, by inhibiting the PI3K/AKT/mTOR signaling pathway and activating the Nrf2/HO-1 pathway, downregulate the expressions of Bax, Caspase-3 and autophagic marker p62, upregulate the key autophagic protein Beclin1 and the LC3-II/I ratio, reduce podocyte apoptosis, enhance autophagosome formation, decrease MDA, increase SOD, GSH and CAT, improve oxidative stress, and synchronously regulate antioxidant capacity and autophagy to delay the progression of MN (Di Tu et al., 2020).

**FIGURE 3:**
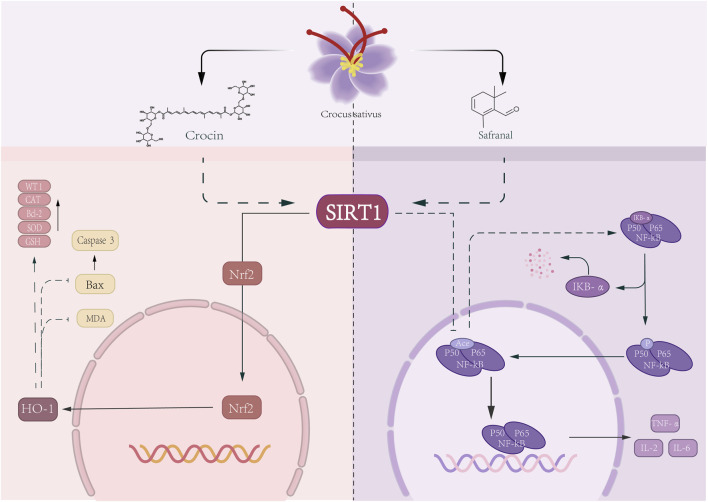
Crocin and Safranal through SIRT1-mediated antioxidant and anti-inflammatory effects CAT: catalase Bcl-2:B cell lymphoma-2 SOD: Superoxide Dismutase GSH: Glutathione Bax:BCL-2-associated X protein MDA: malondialdehyde.

Crocin and Safranal, two distinct key active metabolites in Crocus sativus, both participate in the renal protective process of MN by targeting the regulation of SIRT1 expression. In the PHN rat model, crocin activates SIRT1 through the Nrf2/HO-1 pathway, reduces MDA levels, enhances SOD activity, effectively scavenges ROS, and significantly enhances antioxidant stress resistance (Liu et al., 2023b). Simultaneously, safranal negatively regulates the NF-κB pathway via SIRT1, thereby reducing IL-6 levels (Bao et al., 2025). In addition, two studies in 2023 and 2024 have collectively revealed the renoprotective effect of Crocin through its antioxidant activity. In the PHN rat model, Crocin (100 mg/kg/d via intraperitoneal injection for 30 days) activated the SIRT1/Nrf2/HO-1 pathway, reduced MDA levels, simultaneously enhanced the activities of SOD, GSH, and CAT, alleviated oxidative damage of renal tissue and GBM thickening in MN, decreased the deposition of IgG, C3, and C5b-9, inhibited podocyte apoptosis by downregulating Bax and caspase-3 as well as increasing the number of WT-1-positive podocytes and Bcl-2 expression, and improved SCr, BUN, TC, and urinary protein levels (Liu et al., 2023b). In the gentamicin-induced nephrotoxicity model in rats, crocin (low and high doses of 25 mg/kg and 50 mg/kg, administered via intraperitoneal injection for 8 days) reduces MDA levels by activating the Nrf2/HO-1 signaling pathway, while enhancing the activities of SOD, GSH, and CAT, thereby alleviating oxidative damage to renal tissue in MN. Meanwhile, it inhibits the TLR-4/NF-κB inflammatory pathway by downregulating NF-κB, COX-2, TLR-4, Bax, and Caspase-3, and upregulating Bcl-2, reduces the secretion of TNF-α, IL-1β, and IL-6, decreases the levels of UA, SCr, and BUN, and mitigates the pathological damage of MN (Dogan et al., 2024). While these findings are promising, the doses used in animal studies (e.g., 100 mg/kg crocin) far exceed the achievable plasma concentrations in humans after oral administration of saffron, raising concerns about clinical translatability (Figure 3).

### 2.3 The regulatory role of CHMs in autophagy and apoptosis

#### 2.3.1 Autophagy regulation

Autophagy exerts bidirectional regulatory effects on the development of MN. On the one side, autophagy activates innate and adaptive immunity, promotes autoantibody production and complement activation, thereby amplifying renal injury. On the other side, autophagy protects podocytes from apoptosis, maintains renal function, and counters injury progression (Ponticelli et al., 2025). CHMs for the treatment of MN can alleviate podocyte injury by targeted regulation of autophagy through pathways such as the mTOR/ULK1 pathway, Wnt/β-Catenin pathway, and PINK1/Parkin pathway, providing an important direction for the treatment of MN.

Shenqi Dihuang Decoction (SQDHD) is a classic TCM formula that has undergone long-term clinical practice with a rich historical background. It is composed of *A. mongholicus* Bunge [Fabaceae; Astragali radix], *Rehmannia glutinosa* (Gaertn.) DC. [Orobanchaceae; Rehmanniae radix praeparata], *Dioscorea oppositifolia* L [Dioscoreaceae; Dioscoreae rhizoma], *Smilax glabra* Roxb. [Smilacaceae; Smilacis glabrae rhizoma], *Paeonia* × *suffruticosa* Andrews [Paeoniaceae; Moutan cortex], *Cornus officinalis* Siebold & Zucc. [Cornaceae; Corni fructus], and *P. ginseng* C.A.Mey [Araliaceae; Ginseng radix]. In 2024, Wang et al. demonstrated in PHN rats and MPC-5 cell models that SQDHD (administered via oral gavage at low and high doses of 0.30 g/kg/d and 0.60 g/kg/d for 4 weeks *in vivo*, via drug-containing serum treatment *in vitro*) could inhibit USP14, activate K63-ubiquitination of Beclin1, increase the LC3II/I ratio, downregulate P62 expression, enhance podocyte autophagosome formation, reduce urinary protein levels, and improve MN renal pathological damage (Wang et al., 2025c).

Comprising the botanical drugs *A. membranaceus* (Fisch.) Bunge [Fabaceae; *Astragali radix*], *Angelica polymorpha* Maxim. [Apiaceae; *Angelicae sinensis radix*], *Atractylis chinensis* (Bunge) DC [Asteraceae; *Atractylodis rhizoma*], *C. pilosula* (Franch.) Nannf. [Campanulaceae; *Codonopsis radix*], *A. macrocephala* Koidz [Asteraceae; *Atractylodis macrocephalae rhizoma*], *Poria cocos* (Schw.) Wolf [Polyporaceae; *Poria*], *Coix lacryma-jobi* L. var. *ma-yuen* (Roman.) Stapf [Poaceae; *Coicis semen*], *A. orientale* (Sam.) Juz. [Alismataceae; *Alismatis rhizoma*], *S. miltiorrhiza* Bunge [Lamiaceae; *Salviae miltiorrhizae radix et rhizoma*], *Ligusticum chuanxiong* Hort [Apiaceae; *Chuanxiong rhizoma*], *C. reticulata* Blanco [Rutaceae; *Citri reticulatae pericarpium*], *B. chinense* DC [Apiaceae; *Bupleuri radix*], and *G. uralensis* Fisch [Fabaceae; *Glycyrrhizae radix et rhizoma*], Shenqi Granules (SQG) is a TCM compound formula clinically used specifically for the management of MN. With a decades-long clinical application history in China, its pharmacological effects have been validated through long-term clinical practice (Wei et al., 2023). In 2024, Wei et al. discovered in a PAN-induced MPC-5 injury model that SQG (0.5 mg/mL *in vitro*) could activate autophagy by inhibiting the phosphorylation of mTOR and ULK1, thereby upregulating the expression of podocyte cytoskeletal proteins α-actinin-4 and CD2AP. This reduced podocyte damage and improved MN pathological alterations (Wei et al., 2023).

Classified as a diterpene bioactive metabolite, triptolide is sourced from the classic TCM botanical species *Tripterygium wilfordii* Hook. f. [Celastraceae; Tripterygii wilfordii radix] (Zhang et al., 2024c), exhibiting significant antitumor, anti-inflammatory, and immunosuppressive activities (Hu et al., 2024b). In 2022, Zhang et al. discovered in a PHN rat model that triptolide (200 mg/kg/d administered via gavage for 50 days) could downregulate PI3K, AKT, and mTOR phosphorylation levels, upregulate synaptopodin expression, and downregulate desmin expression. This subsequently increases ALB levels while decreasing TC, TG, 24h-UPE, and LDL levels, improving MN (Zhang et al., 2022).

Additionally, in 2024, Wang et al. demonstrated in PHN rat models and serum-induced primary foot cell injury models that Fangji Huangqi decoction (FJHQ) administered at low and high doses of 0.29 g/kg/d and 0.58 g/kg/d via gavage for 4 weeks *in vivo*, treated with rat serum *in vitro*. Could activate the BNIP3-mediated mitochondrial autophagy pathway by upregulating BNIP3 expression. This pathway downregulated P62 expression, increased LC3B expression and the LC3II/I ratio, enhanced mitochondrial membrane potential, reduced podocyte injury, and thereby improved MN (Wang et al., 2024c).

In 2022, Gao et al. demonstrated in a PHN rat model that MFSD (1 mL/100 g/d administered via gavage for 12 weeks) promoted renal autophagy levels, reduced urinary protein levels, alleviated podocyte injury, and improved renal pathology by regulating autophagy and downregulating Wnt/β-Catenin pathway-related protein expression. Its efficacy was comparable to Cyclosporin A, yet it exhibited superior safety (Gao et al., 2022).

Salvianolic acid B (SalB) is a metabolite of the *S. miltiorrhiza* Bunge [Lamiaceae; Salviae miltiorrhizae radix et rhizoma] (Chen et al., 2023b), exhibiting a bioactivity profile that covers antioxidant, anti-inflammatory, and fibrosuppressive properties (Fu et al., 2024a; Sun et al., 2024). In 2022, Zhou et al. demonstrated in C-BSA-induced Sprague-Dawley rat MN models and lipopolysaccharide-induced human mesangial cell injury models that SalB (100 mg/kg/day orally for 14 days *in vivo*, SalB treatment *in vitro*) exhibited the ability to impede the PI3K/AKT signaling pathway through upregulating miR-145-5p expression, induce autophagy, raise the LC3-II/LC3-I ratio and the expression of Beclin1, reduce mesangial cell hyperplasia plus the secretion of IL-1β, IL-6, and TNF-α, thus improving proteinuria and pathological lesions in renal tissues (Chen et al., 2022).

The key E3 ubiquitin ligase Parkin, as a core molecule for maintaining mitochondrial homeostasis and disease protection, can mediate mitochondrial autophagy through the PINK1-dependent classical pathway. The role of the Jianpi QuShi Huoluo Formula (JQHF) in regulating this pathway and providing renal protection has been confirmed by a series of studies. In 2021, Wang et al. demonstrated in PHN rat model induced by sheep anti-rat Fx1A serum that JQHF (16.2 g/kg/d intragastrically for 4 weeks) could activate the PINK1/Parkin signaling pathway, downregulate the expression of cytoplasmic Parkin, P62, cytochrome c (Cyt c), and Caspase-3, upregulate the expression of PINK1, mitochondrial Parkin, and LC3-II/I, and reduce reactive oxygen species (ROS) production. Additionally, control experiments showed that JQHF exhibited significantly superior therapeutic efficacy compared with the traditional drug Benazepril and the monomer component TET (Wang et al., 2021c). In 2025, Yan et al. demonstrated in PHN rat models and C5b-9-induced podocyte injury models that JQHF (*in vivo*: intragastric administration of low and high doses at 16.2 g/kg/d and 32.4 g/kg/d for 6 weeks; *in vitro*: treatment with drug-containing serum) could directly promote PINK1-dependent mitophagy, thereby activating the PINK1/ROS/NLRP3 signaling pathway, downregulated the expression of NLRP3, caspase-1, desmin, and inflammatory factors, while upregulating the expression of nephrin and podocin, reduced mitochondrial ROS accumulation, improved MMP and mitochondrial ultrastructure, decreased 24h-UPE and TC levels, increased ALB levels, and diminished IgG and C5b-9 deposition, inhibited podocyte injury and alleviated the pathological progression of MN as well as renal function impairment (Yan et al., 2025).

Madecassoside (MA) is a five-ring triterpenoid derived from the botanical drug *Centella asiatica* (L.) Urb. [Apiaceae; Centellae asiaticae herba], a plant widely used in traditional Chinese therapy (Wang et al., 2025a). In 2025, Liu et al. demonstrated in a PHN rat model that MA (30 mg/kg/day administered intraperitoneally for 4 weeks) directly activates AMPK and inhibits mTOR, upregulates Beclin-1 expression and the LC3 II/I ratio, downregulates p62 expression to activate classical autophagy, reduces IgG and C3 deposition, upregulates nephrin, podocin, and synaptopodin expression, downregulated MDA and NO, increased SOD, GSH, GPx, and CAT activity, inhibited TNF-α, IL-1β, IL-6, ICAM-1, VCAM-1, and MCP-1 expression, reduce CD4^+^T, CD8^+^T, and F4/80^+^ infiltration, decrease 24h-UPE, TC, TG, SCr, and BUN, increase ALB, and ultimately improve renal injury in experimental membranous nephropathy (Liu et al., 2025a).

#### 2.3.2 Apoptosis regulation

In the pathological process of MN, podocyte apoptosis serves as a pivotal component. Acting as the initial impetus in a “domino effect,” it triggers characteristic pathological alterations and directly disrupts the core structures of the glomerular filtration barrier. Manifestations include podocyte foot process fusion, disappearance, downregulation of slit membrane protein expression, deposition of electron-dense material in the outer GBM, and widespread fusion of epithelial podocytes (Zhang et al., 2023; Hu et al., 2021). Apoptosis can also trigger cytoskeletal remodeling, such as shortening and disorganized arrangement of F-actin filaments, further exacerbating dysfunction of the filtration barrier (Li et al., 2022b). Simultaneously, inflammatory mediators released during apoptosis recruit macrophages to infiltrate the renal interstitium, accompanied by vacuolar degeneration of tubular epithelial cells (Ke et al., 2023). Furthermore, prolonged podocyte apoptosis can lead to glomerulosclerosis (Hu et al., 2021).

Wenyang Lishui Decoction (WYD) is derived from the classical formula ZWT by adjusting the dosage of certain botanical drugs and adding *A. membranaceus* (Fisch.) Bge. [Fabaceae; Astragali radix]. It consists of *A. carmichaelii* Debx [Ranunculaceae; Aconiti lateralis radix praeparata], *A. membranaceus* (Fisch.) Bunge [Fabaceae; Astragali radix], *Poria cocos* (Schw.) Wolf [Polyporaceae; Poria], *A. macrocephala* Koidz. [Asteraceae; Atractylodis macrocephalae rhizoma], *P. lactiflora* Pall [Paeoniaceae; Paeoniae radix alba] and *Z. officinale* Rosc. [Zingiberaceae; Zingiberis rhizoma recens]. In 2020, Lu et al. demonstrated in C-BSA-induced MN rat models and mouse podocyte injury models induced by MN patient serum that WYD (administered via oral gavage at 16.5 g/kg/day for 4 weeks *in vivo*, via serum treatment *in vitro*) downregulated p53, upregulated Bcl-2, inhibited podocyte apoptosis, reduced 24 h-UPE levels, improved GBM thickening and podocyte foot process fusion, and mitigated renal tissue damage in MN (Lu et al., 2020).

In 2021, Wang et al. demonstrated in passive PHN rat models and 400 ng/mL doxorubicin hydrochloride (ADR)-injured apoptotic podocyte model that SQ (administered via oral gavage at low and high doses of 6.3 mg/kg/d and 12.6 mg/kg/d for 21 days, treated *in vitro* at 600 μg/mL) upregulating HO-1 and Bcl-2 expression, downregulating Caspase-3 and Bax expression, reducing TUNEL-positive podocyte numbers, inhibiting podocyte apoptosis and GBM thickening, and decreasing proteinuria (Wang et al., 2021a).

Kemenfang (KMF) is a TCM compound consisting of 13 botanical drugs. In 2024, Li et al. demonstrated in a C-BSA-induced MN rat model that KMF (administered via oral gavage at low, medium, and high doses of 3.3075 g/kg/day, 6.615 g/kg/day, and 13.23 g/kg/day for 4 weeks) upregulates PI3K, AKT phosphorylation levels, promotes Bcl-2 and p-BAD expression, downregulates BAX and Cleaved Caspase-3 expression, restores WT-1 and Nephrin levels, mitigates GBM thickening and mitochondrial swelling, and reduces TUNEL-positive apoptotic cells (Li et al., 2024). However, unlike KMF, which activates the PI3K/AKT pathway to enhance the anti-apoptotic capacity of foot cells, Tetrandrine represents a natural dibenzylideneisoquinoline alkaloid obtained from *Stephania tetrandra* S. Moore [Menispermaceae; Stephaniae tetrandrae radix], an botanical drug integral to TCM formulations (Ji et al., 2024; Tan et al., 2024), By suppressing the PI3K/AKT signaling cascade, this compound markedly diminishes the levels of p-PI3K and p-AKT, abrogates BAD-mediated inhibition of Bcl-2, elevates the expression of Bcl-2 and p-BAD, and concurrently downregulates the expression of BAX and Cleaved Caspase-3, thereby mitigating podocyte apoptosis in MN (Yin et al., 2021).

**FIGURE 4:**
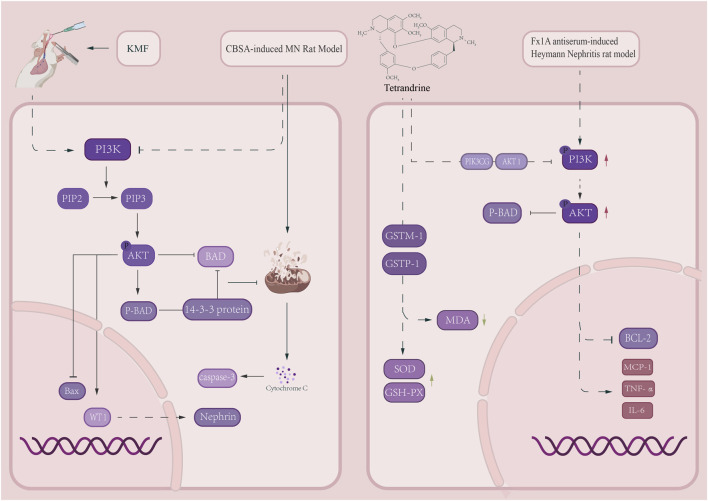
PI3K: Tetrandrine and Kemengfang Exert Opposite Regulation on PI3K and AKT Phosphorylation and Their Renal Protective Mechanisms phosphatidylinositol 3-kinase AKT: protein kinase B BAD:bcl-2 agonist of cell death.

Although Tetrandrine and KMF exhibit opposite regulatory directions on PI3K and AKT phosphorylation, both achieve podocyte protection by modulating the BCL-2/BAD/Caspase-3 apoptotic axis. This “bidirectional regulation” characteristic may correlate with pathway activity differences across distinct pathological stages of MN. Together, they validate the efficacy and precision-regulation advantages of CHMs in treating MN (Figure 4).

#### 2.4 The podocyte protection and anti-fibrotic effects of CHMs

Renal fibrosis represents the ultimate outcome of advanced MN (Fu et al., 2024b), and its development is closely associated with multiple cellular and molecular mechanisms. In combating renal fibrosis, renal tubular epithelial cells activate the MAPK/ERK-EGR1-FGF2 signaling axis to promote the secretion of Fibroblast Growth Factor 2 (FGF2). This paracrine action activates fibroblasts and drives extracellular matrix deposition (Livingston et al., 2024). Further studies have revealed that injured renal tubular epithelial cells directly activate pericytes and myofibroblasts through epithelial-mesenchymal crosstalk and exacerbate the inflammatory microenvironment. Among these mechanisms, TGF-β induces the expression of α-smooth muscle actin (α-SMA) via the classical Smad signaling pathway, driving the transition of fibroblasts to a myofibroblast phenotype (Huang et al., 2023), while the Wnt/β-catenin pathway enhances the process of tissue fibrosis by regulating the synthesis of extracellular matrix (Rieder et al., 2025).

As the primary active metabolite of artemisinin, dihydroartemisinin (DHA) is sourced from Artemisia annua L [Asteraceae; Artemisiae annuae herba], a botanical drug widely used in traditional Chinese botanical medicine practice (Wang et al., 2024a; Wen et al., 2024). It is a key drug for clinical antimalarial therapy, renowned for its high efficacy and rapid onset of action (Rosenthal et al., 2024). Furthermore, the therapeutic potential of DHA extends far beyond the field of antimalarial treatment, as it has been confirmed to specifically treat immune-inflammatory diseases, organ fibrosis, and malignant tumors (Zhu et al., 2025; Dai et al., 2024; Qiu et al., 2024; Jin et al., 2023). In 2023, Li et al. demonstrated in a C-BSA-induced MN mouse model that DHA (10 μM/d administered via gavage for 3 weeks) could inhibit TGF-β1/Smad signaling pathways, thereby downregulating α-SMA and ED-1 expression, upregulate nephrin and podocin expression, reduce IgG and C3 deposition, lower 24-UPE, TC, and TG levels, and improve renal histopathology, inhibits podocyte injury and alleviates renal fibrosis in MN mice (Li et al., 2023b).

MSG not only suppresses MN-related inflammatory responses by regulating the NF-κB/Nrf2 axis (Ma et al., 2023) but also exhibits significant anti-fibrotic effects. In 2023, Wang et al. found in the C-BSA-induced MN rat model and Zymosan-Activated Serum-induced podocyte injury model that MSG (low and high doses of 1.85 g/kg and 3.70 g/kg, intragastric administration for 4 weeks) and its active metabolite AS-IV (10 μM, 20 μM, 40 μM) could downregulate the expression of fibrosis-related proteins such as MMP-7, PAI-1, and FSP1 by inhibiting the Wnt1/β-catenin pathway, while suppressing the expression of key molecules in the RAS pathway including AGT, ACE, and angiotensin II type 1 receptor (AT1R), upregulating the expression of podocin and nephrin, and reducing glomerulosclerosis and renal tubulointerstitial fibrosis in MN (Wang et al., 2023c).

Rhodojaponin VI (RJ-VI) is one of the most representative grayanane diterpenoids, featuring a unique 5/7/6/5 tetracylic skeleton. It is widely distributed in the flowers, fruits, and roots of Rhododendron molle, a plant belonging to the Ericaceae family (Song et al., 2025). In 2024, Song et al. demonstrated in PHN rat models and C5b-9-induced human progenitor cell injury models that RJ-VI (0.02 mg/kg/d orally for 4 weeks *in vivo*; *in vitro* concentration not specified) could inhibit MDM2 expression, block the MDM2/Notch1 signaling pathway, downregulate desmin expression, and upregulate nephrin, WT-1, and podocin expression, reduced immune complex deposition and podocyte foot process fusion, thereby alleviating proteinuria and glomerular damage while delaying MN progression (Song et al., 2024).

Sanqi Qushi Formula (SQQS) has demonstrated multi-target protective mechanisms in preventing and treating renal injury, along with favorable clinical efficacy (Lin et al., 2025). In 2025, Lin et al. found in PHN rat models and TNF-α-induced podocyte injury models that SQQS (intragastric administration at 12.6 and 25.2 g/kg/d for 3 weeks *in vivo*; *in vitro* treatment with its active metabolite Methylnissolin-3-O-glucoside at concentrations of 1.5, 5, and 15 μM) exerts podocyte protective effects and delays renal fibrosis. This is achieved by directly binding to MEK1, inhibiting the MEK/ERK signaling pathway, blocking TNF-α-induced EMT, reducing CD68^+^ macrophage infiltration, ameliorating GBM thickening, and alleviating the persistent damage to podocytes caused by immune complex deposition (Lin et al., 2025). In the same year, Sun et al. found in the PHN rat model and the zymosan-activated serum (ZAS, 10% v/v)-induced mouse immortalized podocyte MPC-5 injury model that SQQS (*in vivo*: intragastric administration of low, medium, and high doses at 5.3 g/kg/d, 10.6 g/kg/d, 21.2 g/kg/d for 3 weeks; *in vitro*: treatment with 5%, 10%, 15% dose SQQS-containing rat serum HS) could downregulate the expression of endoplasmic reticulum stress (ERS)-related markers such as p-PERK and CHOP, as well as ferroptosis-promoting proteins including ACSL4 and CHAC1, upregulate antioxidant and ferroptosis-inhibiting proteins such as GPX4 and xCT, inhibit ERS-induced ferroptosis, upregulate the expression of critical podocyte structural proteins namely Nephrin, Podocin and WT-1, suppress desmin expression, and decrease iron deposition as well as ROS and MDA content in renal tissues, restore GSH content and SOD activity, decrease C3 deposition, inhibit podocyte injury and glomerular fibrosis, and ameliorate renal function in MN (Sun et al., 2025).

It is particularly noteworthy that the synergistic effects between *A. membranaceus* (Fisch.) Bge. [Fabaceae; Astragali radix] and *P. notoginseng* (Burk.) F.H. Chen [Araliaceae; Notoginseng radix et rhizoma], as the core botanical drugs of the SQQS, have been further validated. In 2025, Wang et al. found in the PHN rat model and ADR (400 ng/mL)-induced podocyte injury model that SQ, composed solely of Astragalus and Panax notoginseng (*in vivo*: intragastric administration of low and high doses at 6.3 mL/kg and 12.6 mL/kg for 3 weeks; *in vitro*: treatment with 600 μg/mL), could inhibit EMT by regulating the ERK/CK2-α/β-catenin signaling pathway, downregulate vimentin and α-SMA, while upregulate Synaptopodin and Podocin, reduce the number of apoptotic podocytes, and alleviate podocyte injury induced by MAC and C5b-9. Thereby, it protects podocytes and delays the progression of glomerular fibrosis in MN (Wang et al., 2025b).

#### 2.5 Microbiome and immunomodulatory effects of CHMs

In the pathophysiology of MN, gut microbiota dysbiosis plays a pivotal role by disrupting intestinal homeostasis and exhibits bidirectional regulation with MN (Shang et al., 2022; Shi et al., 2023). Mendelian randomization analysis indicates that increased abundance of gut bacterial genera such as Alistipes, Butyricicoccus, and Butyrivibrio reduces the risk of MN disease, whereas genera like Oscillibacter increase the risk of MN disease (Qing et al., 2025). Dysbiosis of the oral microbiota is also closely associated with the pathological progression of MN. Compared to healthy individuals, the composition and structure of the oral microbiota in MN patients exhibit significant abnormalities. Changes in bacterial genus abundance correlate closely with clinical indicators of MN (Luan et al., 2022). In addition, the dysbiosis of the salivary microbiota can interact with the gut microbiota through immunoinflammatory mechanisms, jointly participating in the occurrence and progression of MN (Ramanathan et al., 2023).

**FIGURE 5:**
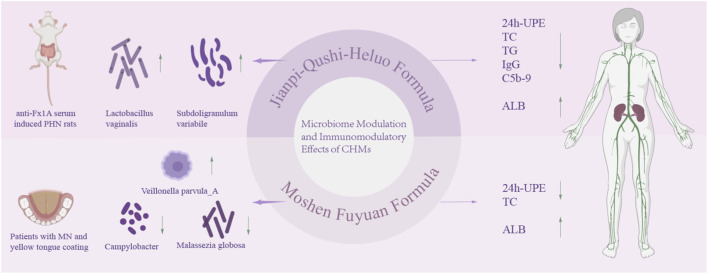
Microbiome and Immunomodulatory Effects of CHMs 24h-UPE:24-h urine protein excretion TC: total cholesterol TG: Triglycerides ALB: serum albumen.

From 2022 to 2024, Wang et al. conducted a series of studies on the microbiological regulatory mechanisms of TCM formulations in treating MN. In the anti-Fx1A antiserum-induced PHN rat model, JQHF (administered via gavage at low and high doses of 16.2 g/kg/d and 32.4 g/kg/d for 4 weeks) significantly enriched *Lactobacillus* vaginalis and Subdoligranulum variabile by modulating gut microbiota composition. This reduced IgG and C5b-9 deposition, inhibited glomerular and tubular cell apoptosis, lowered 24h-UPE, TC, and TG levels, elevated ALB, and improved renal tissue pathological damage (Wang et al., 2022b). Studies on MN patients indicate that Moshen Fuyuan Formula (MSFY) exerts therapeutic effects by targeting the oral microbiome. MSFY (1 sachet twice daily orally for 12 weeks) downregulates the abundance of Campylobacter—a characteristic bacterium in yellow plaque patients—and the pro-inflammatory fungus Malassezia globosa, while upregulating Veillonella parvula_A expression. By balancing the dynamic interactions between oral bacteria and fungi, it also achieves reductions in 24h-UPE and TC alongside increases in ALB (Wang et al., 2024b). Both findings collectively demonstrate that TCM formulas can improve renal function indicators in MN by regulating the gut and oral microbiomes, providing evidence for the role of the “microbiome-kidney axis” in the treatment of this disease. Although this study did not specifically elucidate the precise mechanisms by which TCM affects the gut microbiota in MN, previous research indicates that gut microbiota can participate in the pathogenesis of MN through multiple pathways. Protective gut microbiota, such as Ruminococcaceae UCG003 and *Streptococcus*, can inhibit the release of inflammatory factors, including IL-6, TNF, and IL-1β, block the IL-17/TNF signaling pathway, enhance intestinal barrier function by producing short-chain fatty acids (SCFAs), and regulate SIgA to maintain the immune homeostasis of the intestinal-kidney axis. In contrast, risk-associated gut microbiota such as Oscillibacter promote the expression of the inflammatory factors, activate the Toll-like receptor pathway, and exacerbate kidney injury. Furthermore, derivatives of tryptophan metabolized by gut microbiota can exert a protective effect on MN by inhibiting the AHR pathway (Qing et al., 2025) (Figure 5 and Tables 1, 2).

**TABLE 1:** The mechanisms of CHMs to treat MN.

Mechanism	Molecular pathway	Botanical drug prescription or the active metabolite’s name	References	*In vivo* models	Outcomes	*In vitro* models	Outcomes
Anti-inflammatory and Immunomodulatory Effects of CHMs	NF-κB signaling pathway	Zhen-Wu-Tang Decoction	Liu et al. (2019)	C-BSA-induced MN Rat Model	Decrease:NLRP3,Caspase-1,IL-1β,p-p65,p-IκBα	TNF-α-induced podocyte injury model	Decrease:NLRP3,Caspase-1,IL-1β,NF-κB p-p65,p-IκBα
	​	Diosgenin	Jia et al. (2024)	C-BSA-induced MGN Rat model	Decrease: MDA,Keap1,IL-2,TNF-α,IL-6,NF-κB p65 Increase:HO-1,Nrf2	​	​
	​	Sanqi oral solution	Tian et al. (2019)	C-BSA-induced MN rat model	Decrease:NF-κB p65,NF-κB p-p65	​	​
	​	Modified Huangqi Chifeng decoction	Chang et al. (2025)	Sheep anti-rat Fx1A serum induced the PHN rat model	Decrease:p-NF-κB p65 Increase: WT-1	​	​
	​	Moshen granule	Ma et al. (2023)	IMN patient CBSA-induced MN Rat Model	Decrease: AHR,NF-κB p65 Increase:Nrf2,HO-1	​	​
	​	Astragaloside IV	Ma et al. (2024)	PHN rat model	Decrease: TNF-α,IL-6,IL-1β	TNF-α-induced MPC5 podocytes	Decrease:TNF-α,IL-6,IL-1β, TRAF6, p-NF-κB
	​	Safranal	Bao et al. (2025)	CBSA-induced MGN Rat Model	Decrease:NF-κB p-p65,p-IκB,IL-6,TNF-α Increase:WT-1,Sirt1	​	​
	JAK-STAT signaling pathway	Shengyang Yiwei Decoction	Wu et al. (2025)	PHN rat model	Increase:IFN-γ,T-bet,Th1 Decrease:IL-4,GATA3,p-JAK2/JAK2,TH2,p-STAT3/STAT3	​	​
	​	Colquhounia Root Tablet	Mao et al. (2023)	C-BSA-induced MN rat model	Decrease:TNF-α,IL-6,MMP9,p-JAK2,p-STAT3	C5b-9-injured MPC5 podocytes	Decrease:TNF-α,IL-6,MMP,p-JAK2,p-STAT3
	IL-6/STAT3 signal pathway	Mahuang Fuzi and Shenzhuo Decoction	Zhao et al. (2024)	MN patients PHN rat model	Decrease: IL-6 in MN patients Decrease:IL-6,IL-4,IFN-γ,pSTAT3 Increase: Bcl-2 in the PHN rat model	​	​
	Complement activation pathway	Total coumarin derivatives from Hydrangea paniculata	Wang et al. (2022a)	C-BSA-induced MN rat model	Decrease: IL-10,CD20,α-SMA,p-AKT,C3b,C3,p-PI3K,p-NF-kB	​	​
	​	polyamide resin of corn silk Ethanol extract	Wang et al. (2021b)	C-BSA-induced MN rat model	Decrease: IgG,C3	​	​
	​	Yi Shen An capsules	Zhao et al. (2022)	C-BSA-induced MN rat model	Decrease: CIC,IgM,C3c Increase: IgG	​	​
	Th17/Treg Immunomodulatory Axis	QingreHuoxue formula	Lou et al. (2024)	C-BSA-induced MN mouse model	Decrease:Th17cells, IL-17,IgG,TGF-β1 Increase:Treg cells	​	​
The Antioxidant Effects of CHMs	Nrf2/HO-1 Signaling Pathway	Curcumin	Di Tu et al. (2020)	anti-Fx1A antiserum-induced PHN rat model	Decrease:PI3K,p-AKT,p-mTOR Increase: Nrf2,HO-1,LC3-II/I	​	​
	​	Crocin	Liu et al. (2023b) Dogan et al. (2024)	anti-Fx1A antiserum-induced PHN rat model Gentamicin-induced nephrotoxicity model	Increase: Sirt1, Nrf2,HO-1 in anti-Fx1A antiserum-induced PHN rat model Decrease: TNF-α,IL-1β,IL-6,TLR-4,NF-κB Increase:SOD,GSH,Nrf-2,HO-1 in Gentamicin-induced nephrotoxicity model	​	​
The Regulatory Role of CHMs in Autophagy and Apoptosis	USP14-Beclin1-autophagy pathway	Shen-Qi-Di-Huang Decoction	Wang et al. (2025c)	PHN rat model	Decrease: USP14,P62Increase: LC3-II/LC3-I ratio, Beclin1	MPC5 cell model induced by serum from rats	Decrease:USP14,P62Increase:LC3-II/LC3-I ratio, Beclin1,K63-ubiquitinated Beclin1
	mTOR/ULK1 autophagy pathway	Shenqi granule	Wei et al. (2023)	​	​	PAN-induced MPC5 podocyte injury model	Decrease:p-mTOR,p-ULK1 Increase:CD2AP,α-actinin4,
	PI3K/AKT/mTOR signal pathway	Triptolide	Zhang et al. (2022)	PHN rat model	Decrease: p-PI3K,p-AKT,p-mTOR	​	​
	BNIP3-mediated mitophagy	Fangji Huangqi decoction	Wang et al. (2024c)	PHN rat model	Decrease: P62 Increase: BNIP3,LC3B, LC3 II/I ratio	Primary podocytes treated with PHN rat serum	Decrease: P62 Increase:BNIP3,LC3B
	Wnt/β-Catenin signal pathway	Mahuang Fuzi and Shenzhuo Decoction	Gao et al. (2022)	PHN rat model	Decrease:β-catenin,GSK-3β,LC3-II,p62	​	​
	miR-145-5p/PI3K/AKT pathway	Salvianolic acid B	Chen et al. (2022)	C-BSA-induced MN rat model	Decrease: TNF-α,PI3K,p-AKT Increase:Beclin1,miR-145-5p	LPS-induced human mesangial cells	Decrease:IL-1β,IL-6, TNF-α,PI3K,P-AKT Increase:miR-145-5p
	PINK1/Parkin signal pathway	Jianpi Qushi Heluo Formula	Wang et al. (2021c)	anti-Fx1A serum-induced PHN rats	Decrease: Parkin,P62,Cyt c,NLRP3,ROSIncrease: PINK1,LC3-II/I ratio	​	​
	PINK1/ROS/NLRP3 signal pathway	Jianpi Qushi Heluo Formula	Yan et al. (2025)	anti-Fx1A serum-induced PHN rats	Decrease: NLRP3,ROS Increase: PINK1	C5b-9-stimulated podocyte injury model	Decrease: ROS,NLRP3 Increase: PINK1
	AMPK/mTOR signal pathway	Madecassoside	Liu et al. (2025a)	PHN rat model	Decrease: mTOR,p62,TNF-α Increase: AMPK, Beclin1,LC3 II/I ratio	​	​
	p53/Bcl-2-mediated apoptosis signaling pathway	Wenyang Lishui Decoction	Lu et al. (2020)	C-BSA-induced MN Rat Model	Decrease: P53 Increase: Bcl-2	MN patient serum-induced podocyte injury model	Decrease:p53 Increase: Bcl-2
	Nrf2/HO-1 Signaling Pathway	Sanqi oral solution	Wang et al. (2021a)	PHN rat model	Decrease: Bax, Cleaved Caspase-3 Increase:Bcl-2,Nrf2,HO-1	ADR-induced podocyte injury model	Decrease: Cleaved Caspase-3 Increase:Nrf2,HO-1
	PI3K/AKT signal pathway	Kemeng Fang	Li et al. (2024)	CBSA-induced MN Rat Model	Decrease: BAX,c-caspase3 Increase: WT-1,BCL-2,P-BAD,PI3K,P-AKT	​	​
	​	Tetrandrine	Yin et al. (2021)	Fx1A antiserum-induced Heymann Nephritis rat model	Decrease:p-PI3K,p-Akt,Caspase-3 Increase: p-BAD,Bcl-2	​	​
The podocyte protection and anti-fibrotic effects of CHMs	TGF-β1/Smad signal pathway	Dhydroartemisinin	Li et al. (2023b)	C-BSA-induced MN mouse model	Decrease:α-SMA,ED-1,TGF-β1,p-Smad2,p-Smad3	​	​
	Wnt1/β-catenin signal pathway	Moshen granule	Wang et al. (2023c)	CBSA-induced MN Rat Model	Decrease:Wnt1,β-catenin,AGT,ACE,AT1R	​	​
	​	Astragaloside IV	Wang et al. (2023c)	​	​	ZAS-Induced Podocyte Injury Model	Decrease:Wnt1,β-catenin,Snail1,Twist
	MDM2/Notch1 signal pathway	Rhodojaponin VI	Song et al. (2024)	PHN rat model	Decrease: MDM2,Notch1 Increase: WT-1	C5b-9-induced human podocyte model	Decrease:MDM2,Notch1
	MEK/ERK signal pathway	Sanqi Qushi formula	Lin et al. (2025)	PHN rat model	Decrease: p-MEK,p-ERK,EMT,α-SMA	TNF-α-induced podocyte injury model	Decrease: p-MEK,p-ERK
	​	Sanqi Qushi formula	Sun et al. (2025)	PHN rat model	Decrease: GRP78,p-PERK,p-eIF2α,ATF4,CHOP, ACSL4,CHAC1 Increase: WT-1,xCT,GPX4	ZAS-Induced Injury immortalized mouse podocytes MPC-5 model	Decrease: GRP78,p-PERK,p-eIF2α,ATF4,CHOP, ACSL4,CHAC1 Increase: WT-1,xCT,GPX4
	ERK/CK2-α/β-catenin signal pathway	Sanqi oral solution	Wang et al. (2025b)	PHN rat model	Decrease:EMT,vimentin,α-SMA,CK2-α,β-catenin	ADR-induced podocyte injury model	Decrease:EMT,α-SMA,vimentin,CK2-α,β-catenin
Microbiome and Immunomodulatory Effects of CHMs	Gut Microbiota-Immune Regulation Pathway	Jianpi Qushi Heluo Formula	Wang et al. (2022b)	anti-Fx1A antiserum induces the PHN rat model	Decrease: IgG,C5b-9 Increase: *Lactobacillus* vaginalis, Subdoligranulum variabile	​	​
	Oral Microbiota-Fungal Interaction Pathway	Moshen Fuyuan Formula	Wang et al. (2024b)	MN patients	Decrease: *Campylobacter*, Malassezia globosa Increase: Veillonella parvula_A	​	​

**TABLE 2:** Summary of CHMs, active metabolites, dosages, and experimental models for MN treatment.

Botanical drug prescription or the active metabolite’s name	Composition	Dosage range	Minimal active concentration	Study model (*in vitro*/*in vivo*)	Control groups (positive/negative)	Treatment duration	Extract type	References
Zhen-Wu-Tang Decoction	*Aconitum carmichaelii* Debeaux [Ranunculaceae; Aconiti lateralis radix praeparata], *Poria cocos* (Schw.) Wolf [Polyporaceae; Poria], *Atractylodes macrocephala* Koidz. [Asteraceae; Atractylodis macrocephalae rhizoma], *Paeonia lactiflora* Pall. [Paeoniaceae; Paeoniae radix alba], *Zingiber officinale* Roscoe [Zingiberaceae; Zingiberis rhizoma recens]	4.2, 8.4, 16.8 g/kg/day	Not specified	*In vivo* (C-BSA-induced MN Rat Model); *In vitro* (TNF-α-induced podocyte injury model)	Positive: prednisone (2 mg/kg/day); Negative: normal saline/untreated model/TNF-α only	*In vivo*: 4 weeks; *In vitro*: 24 h	Aqueous extract	Liu et al. (2019)
Diosgenin	Diosgenin (purity >98%, cat. no. D1634, Sigma-Aldrich)	10 mg/kg	Not specified	*In vivo* (C-BSA-induced MN Rat Model)	Positive: TPCA1 (10 mg/kg) Negative: distilled water	4 weeks	Pure metabolite (≥98%, commercial source)	Jia et al. (2024)
Sanqi oral solution	*Astragalus membranaceus* (Fisch.) Bge. [Fabaceae; Astragali radix], *Panax notoginseng* (Burk.) F.H. Chen [Araliaceae; Notoginseng radix et rhizoma]	6.3 mL/kg/day	Not specified	*In vivo* (C-BSA-induced MN rat model)	Positive: losartan potassium (10.5 mg/kg/day); Negative: normal saline/untreated model	4 weeks	Aqueous extract (oral solution)	Tian et al. (2019)
Huangqi Chifeng decoction	*Astragalus membranaceus* (Fisch.) Bge [Fabaceae; Astragali radix], *Euryale ferox* Salisb. [Nymphaeaceae; Euryales semen], *Rosa laevigata* Michx. [Rosaceae; Rosae laevigatae fructus], *Paeonia lactiflora* Pall. [Paeoniaceae; Paeoniae radix rubra], *Saposhnikovia divaricata* (Turcz. ex Ledeb.) Schischk. [Apiaceae; Saposhnikoviae radix] , *Hedyotis diffusa* Willd. [Rubiaceae; Hedyotis diffusae herba], *Dioscorea nipponica* Makino [Dioscoreaceae; Dioscoreae nipponicae rhizoma]	12.5 g/kg/day	Not specified	*In vivo* (Sheep anti-rat Fx1A serum induced the PHN rat model)	Positive: benazepril (10 mg/kg/day); Negative: normal saline/untreated model	6 weeks	MSG	Chang et al. (2025)
Moshen granule	*Astragalus membranaceus* (Fisch.) Bunge [Fabaceae; Astragali radix], *Atractylodes macrocephala* Koidz. [Asteraceae; Atractylodis macrocephalae rhizoma], *Eucommia ulmoides* Oliv. [Eucommiaceae; Eucommiae cortex], *Plantago asiatica* L. [Plantaginaceae; Plantaginis semen], *Lycium chinense* Mill. [Solanaceae; Lycii fructus], *Cryptotympana pustulata* Walker [Cicadidae; Cicadae periostracum], *Euonymus alatus* (Thunb.) Siebold [Celastraceae; Euonymi alati ramulus], *Sinomenium acutum* (Thunb.) Rehder and E.H.Wilson [Menispermaceae; Sinomenii caulis], *Dioscorea nipponica* Makino [Dioscoreaceae; Dioscoreae nipponicae rhizoma], *Patrinia scabiosifolia* Link [Caprifoliaceae; Patriniae herba], *Rosa laevigata* Michx. [Rosaceae; Rosae laevigatae fructus], *Schisandra chinensis* (Turcz.) Baill. [Schisandraceae; Schisandrae chinensis fructus], *Eupolyphaga sinensis* Walker [Polyphagidae; Eupolyphaga], *Angelica sinensis* (Oliv.) Diels [Apiaceae; Angelicae sinensis radix]	Human: 40 g/day Rat: 3.70 g/kg (oral)	Not specified	*In vivo*: (human patients with IMN and C-BSA-induced MN rats)	Human: before-treatment baseline Rat: healthy control rats and untreated MN rats	Human: 12 months Rat: 4 weeks	Traditional Chinese patent medicine (granule)	Ma et al. (2023)
Astragaloside IV	AS-IV [purity ≥ 98%] isolated from *Astragalus membranaceus* (Fisch.) Bge. [Fabaceae; Astragali radix]	*In vitro*: 25, 50, 100 μM; *In vivo*: 20, 40 mg/kg/d	Not specified	*In vitro*: (TNF-α-induced MPC5 podocytes); *In vivo*: (PHN rat model)	Positive: Prednisone (2 mg/kg/d); Negative: saline/vehicle (double-distilled water)	*In vitro*: 24 h; *In vivo*: 4 weeks	Pure metabolite (Astragaloside IV, ≥98% purity, Macklin)	Ma et al. (2024)
Safranal	Safranal (from *Crocus sativus* L. [Iridaceae])	100, 200 mg/kg/day	Not specified	*In vivo* (C-BSA-induced MGN Rat Model)	Negative: normal saline/untreated model	4 weeks	Pure metabolite (Safranal)	Bao et al. (2025)
Shengyang Yiwei Decoction	*Astragalus membranaceus* (Fisch.) Bge. [Fabaceae; Astragali radix], *Panax ginseng* C.A.Mey. [Araliaceae; Ginseng Radix], *Atractylodes macrocephala* Koidz. [Asteraceae; Atractylodis Macrocephalae Rhizoma], *Poria cocos* (Schw.) Wolf [Polyporaceae; Poria], *Paeonia lactiflora* Pall. [Paeoniaceae; Paeoniae Radix Alba], *Pinellia ternata* (Thunb.) Breit. [Araceae; Pinelliae Rhizoma], *Alisma orientale* (Sam.) Juz. [Alismataceae; Alismatis Rhizoma], *Glycyrrhiza uralensis* Fisch. [Fabaceae; Glycyrrhizae Radix et Rhizoma Praeparata], *Citrus reticulata* Blanco [Rutaceae; Citri Reticulatae Pericarpium], *Notopterygium incisum* Ting ex H.T.Chang [Apiaceae; Notopterygii Rhizoma et Radix], *Angelica pubescens* Maxim. [Apiaceae; Angelicae Pubescentis Radix], *Saposhnikovia divaricata* (Turcz.) Schischk. [Apiaceae; Saposhnikoviae Radix], *Bupleurum chinense* DC. [Apiaceae; Bupleuri Radix], *Coptis chinensis* Franch. [Ranunculaceae; Coptidis Rhizoma], *Zingiber officinale* Roscoe [Zingiberaceae; Zingiberis Rhizoma Recens], *Ziziphus jujuba* Mill. [Rhamnaceae; Jujubae Fructus]	12 g/kg/day	Not specified	*In vivo* (PHN rat model)	Positive: JAK2 inhibitor (baricitinib, 2 mg/kg/day), STAT3 inhibitor (stattic, 3.75 mg/kg/every other day) Negative: normal control rats, model group rats	6 weeks	Traditional Chinese medicine decoction (granule form)	Wu et al. (2025)
Colquhounia Root Tablet	*Tripterygium hypoglaucum* (Levl.) Hutch [Celastraceae; Tripterygii hypoglaucae radix] (peeled root), formulated as Colquhounia Root Tablet (Chinese patent medicine, SFDA approval number: Z20027411, chemically characterized by UPLC-Q-TOFMS)	*In vitro*: 186, 372, 744 μg/mL; *In vivo*: low, medium, high doses (exact doses not specified)	Not specified	*In vitro*: (C5b-9-induced MPC5 mouse podocytes); *In vivo*: (C-BSA-induced MN rat model)	Positive: prednisone; Negative: normal control, model (vehicle)	*In vitro*: 24 h; *In vivo*: 4 weeks	Tablet (Chinese patent medicine)	Mao et al. (2023)
Mahuang Fuzi and Shenzhuo Decoction	*Ephedra sinica* Stapf [Ephedraceae; Ephedrae herba], *Aconitum carmichaelii* Debx. [Ranunculaceae; Aconiti lateralis radix praeparata], *Glycyrrhiza uralensis* Fisch. [Fabaceae; Glycyrrhizae radix et rhizoma praeparata cum melle], *Zingiber officinale* Rosc. [Zingiberaceae; Zingiberis rhizoma], *Poria cocos* (Schw.) Wolf [Polyporaceae; Poria], *Atractylodes macrocephala* Koidz. [Asteraceae; Atractylodis macrocephalae rhizoma]	1 mL/100 g/d	Not specified	MN patients PHN rat model	Positive: Cyclosporine A; Negative: Normal saline/untreated model	12 weeks	Formula granules (aqueous extract)	Zhao et al. (2024)
Total coumarin derivatives from Hydrangea paniculata	*Hydrangea paniculata* Siebold [Hydrangeaceae; Hydrangeae Paniculatae Folium et Flos] total coumarin derivatives (standardized to contain 70%–75% total coumarin glycosides; major metabolites: skimmin and apiosylskimmin, 50%–55% of total)	7.5, 15, 30 mg/kg/day	Not specified	*In vivo*: (C-BSA-induced MN rat model)	Positive: Mycophenolate mofetil (20 mg/kg/day) Negative: sham control rats, vehicle-treated MN rats	9 weeks	Standardized plant extract (total coumarin derivatives)	Wang et al. (2022a)
polyamide resin of corn silk Ethanol extract	*Zea mays* L. [Poaceae; Maydis stigma] CSEE and its polyamide resin-purified flavonoid-rich extract (PR-CSEE, total flavonoid content 57.4%). Main flavonoids identified by LC-MS include apigenin and derivatives	*In vivo*: 20 mg/kg BW (based on total flavonoid content) for both CSEE and PR-CSEE	Not specified	*In vivo*: (C-BSA-induced MN rat model)	Positive: losartan (25 mg/kg); Negative: normal saline, vehicle	4 weeks	CSEE: crude ethanol extract; PR-CSEE: flavonoid-enriched extract (polyamide resin purification)	Wang et al. (2021b)
Yi Shen An capsules	*Panax notoginseng* (Burkill) F.H. Chen [Araliaceae; Notoginseng Radix et Rhizoma], *Rheum officinale* Baill. [Polygonaceae; Rhei Radix et Rhizoma], *Salvia miltiorrhiza* Bunge [Lamiaceae; Salviae Miltiorrhizae Radix et Rhizoma], *Lonicera confusa* (Sweet) DC. [Caprifoliaceae; Lonicerae Japonicae Flos], *Carthamus tinctorius* L. [Compositae; Carthami Flos], *Forsythia suspensa* (Thunb.) Vahl [Oleaceae; Forsythiae Fructus], *Scutellaria barbata* D. Don [Labiatae; Scutellariae Barbatae Herba], *Wolfiporia extensa* (Peck) Ginns [Polyporaceae; Poria], *Glycyrrhiza uralensis* Fisch. [Leguminosae; Glycyrrhizae Radix et Rhizoma]	0.25, 0.5, 1, 2 g/kg/day	Not specified (≥1 g/kg effective)	*In vivo* (C-BSA-induced MN rat model)	Positive: prednisone acetate (5 mg/kg/day); Negative: normal saline/untreated model	35 days	Aqueous extract (capsule)	Zhao et al. (2022)
QingreHuoxue formula	*Codonopsis pilosula* (Franch.) Nannf. [Campanulaceae; Codonopsis Radix], *Atractylodes macrocephala* Koidz. [Asteraceae; Atractylodis macrocephalae Rhizoma], *Salvia miltiorrhiza* Bunge [Lamiaceae; Salviae miltiorrhizae Radix et Rhizoma], *Hedyotis diffusa* Willd. [Rubiaceae; Hedyotidis Herba], *Scutellaria baicalensis* Georgi [Lamiaceae; Scutellariae Radix], *Pyrrosia lingua* (Thunb.) Farw. [Polypodiaceae; Pyrrosiae Folium], *Plantago asiatica* L. [Plantaginaceae; Plantaginis Herba], *Polyporus umbellatus* (Pers.) Fr. [Polyporaceae; Polyporus], *Angelica sinensis* (Oliv.) Diels [Apiaceae; Angelicae sinensis Radix], *Leonurus japonicus* Houtt. [Lamiaceae; Leonuri Herba] and *Astragalus mongholicus* Bunge [Fabaceae; Astragali Radix]	19.44 g/kg/day	Not specified	*In vivo*: (C-BSA-induced MN rat model)	Positive: prednisone acetate (0.045 mg/kg/day) + cyclosporine A (25 mg/kg/day) Negative: sham control, IMN model (normal saline)	4 weeks	Traditional Chinese medicine decoction	Lou et al. (2024)
Curcumin	Curcumin [1,7-bis(4-hydroxy-3-methoxyphenyl)-1,6-heptadiene-3,5-dione] from *Curcuma longa* L. [Zingiberaceae] (purity not specified; Santa Cruz, SC-200509)	300 mg/kg/d	Not specified	*In vivo*: (anti-Fx1A antiserum-induced PHN rat model)	Positive: none; Negative: normal control (healthy), model (0.5% CMC-Na vehicle)	30 days	Pure metabolite	Di Tu et al. (2020)
Crocin	Crocin (purity 99.41%), sourced from *Crocus sativus* L. [Iridaceae] and *Gardenia jasminoides* J.Ellis [Rubiaceae]	100 mg/kg/day	Not specified	*In vivo*: (anti-Fx1A antiserum-induced PHN rat model)	Positive: enalapril (40 mg/kg/day, i.p.) Negative: sham control, PHN model (distilled water)	30 days	Pure metabolite	Liu et al. (2023b)
Crocin	Crocin from *Crocus sativus* L. [Iridaceae]	25, 50 mg/kg/day	Not specified	*In vivo* (Gentamicin-induced nephrotoxicity model)	Positive: gentamicin; Negative: normal saline/untreated model	8 days	Pure metabolite	Dogan et al. (2024)
Shen-Qi-Di-Huang Decoction	*Astragalus mongholicus* Bunge [Fabaceae; Astragali radix], *Rehmannia glutinosa* (Gaertn.) DC. [Orobanchaceae; Rehmanniae radix praeparata], *Dioscorea oppositifolia* L. [Dioscoreaceae; Dioscoreae rhizoma], *Smilax glabra* Roxb. [Smilacaceae; Smilacis glabrae rhizoma], *Paeonia* × *suffruticosa* Andrews [Paeoniaceae; Moutan cortex], *Cornus officinalis* Siebold & Zucc. [Cornaceae; Corni fructus], *Panax ginseng* C.A.Mey. [Araliaceae; Ginseng radix]	*In vivo*: 0.30, 0.60 g/kg/d; *In vitro*: 10% rat serum from treated rats	Not specified	*In vivo*: (PHN rat model) *In vitro*: (MPC5 cell model induced by serum from rats)	Positive: prednisone (2 mg/kg/d); Negative: normal control (saline), model (vehicle)	*In vivo*: 4 weeks; *In vitro*: 48 h	Water decoction (concentrated to 1 g crude drug/mL)	Wang et al. (2025c)
Shenqi granule	*Astragalus membranaceus* (Fisch.) Bunge [Fabaceae; *Astragali radix*], *Angelica polymorpha* Maxim. [Apiaceae; *Angelicae sinensis radix*], *Atractylis chinensis* (Bunge) DC [Asteraceae; *Atractylodis rhizoma*], *Codonopsis pilosula* (Franch.) Nannf. [Campanulaceae; *Codonopsis radix*], *Atractylodes macrocephala* Koidz. [Asteraceae; *Atractylodis macrocephalae rhizoma*], *Poria cocos* (Schw.) Wolf [Polyporaceae; *Poria*], *Coix lacryma-jobi* L. var. *ma-yuen* (Roman.) Stapf [Poaceae; *Coicis semen*], *Alisma orientale* (Sam.) Juz. [Alismataceae; *Alismatis rhizoma*], *Salvia miltiorrhiza* Bunge [Lamiaceae; *Salviae miltiorrhizae radix et rhizoma*], *Ligusticum chuanxiong* Hort. [Apiaceae; *Chuanxiong rhizoma*], *Citrus reticulata* Blanco [Rutaceae; *Citri reticulatae pericarpium*], *Bupleurum chinense* DC. [Apiaceae; *Bupleuri radix*], and *Glycyrrhiza uralensis* Fisch. [Fabaceae; *Glycyrrhizae radix et rhizoma*]	0.5 mg/mL	Not specified	*In vitro*: (PAN-induced MPC5 podocyte injury model)	Positive: Cyclosporine A (CsA, 0.5 μg/mL) Negative: untreated control, PAN model group	24 h	Traditional Chinese medicine granule	Wei et al. (2023)
Triptolide	Triptolide (from *Tripterygium wilfordii* Hook f. [Celastraceae])	200 μg/kg/day	Not specified	*In vivo* (PHN rat model)	Positive: FK506 (1 mg/kg/day); Negative: normal saline/untreated model	50 days	Pure metabolite	Zhang et al. (2022)
Fangji Huangqi decoction	*Stephania tetrandra S. Moore [Menispermaceae; Stephaniae tetrandrae radix], Astragalus mongholicus Bunge [Fabaceae; Astragali radix], Glycyrrhiza uralensis Fisch. [Fabaceae; Glycyrrhizae radix et rhizoma praeparata cum melle], Atractylodes macrocephala Koidz. [Asteraceae; Atractylodis macrocephalae rhizoma], Zingiber officinale Roscoe [Zingiberaceae; Zingiberis rhizoma recens], Ziziphus jujuba Mill. [Rhamnaceae; Ziziphi jujubae fructus]*	*In vivo*: 0.29, 0.58 g/kg/day; *In vitro*: 10% rat serum	Not specified	*In vivo* (PHN rat model); *In vitro* (primary rat podocytes with model rat serum)	Positive: prednisone (2 mg/kg/day); Negative: normal saline/untreated model/control rat serum	4 weeks	Aqueous extract	Wang et al. (2024c)
Mahuang Fuzi and Shenzhuo Decoction	*Ephedra sinica* Stapf [Ephedraceae; Ephedrae herba], *Aconitum carmichaelii* Debx. [Ranunculaceae; Aconiti lateralis radix praeparata], *Zingiber officinale* Roscoe [Zingiberaceae; Zingiberis rhizoma], *Poria cocos* (Schw.) Wolf [Polyporaceae; Poria], *Atractylodes macrocephala* Koidz. [Asteraceae; Atractylodis macrocephalae rhizoma], *Glycyrrhiza uralensis* Fisch. [Fabaceae; Glycyrrhizae radix et rhizoma]	1 mL/100 g/day	Not specified	*In vivo* (PHN rat model)	Positive: Cyclosporine A (25 mg/kg/day); Negative: normal saline/untreated model	12 weeks	Aqueous extract	Gao et al. (2022)
Salvianolic acid B	*Salvia miltiorrhiza* Bunge [Lamiaceae; Salviae miltiorrhizae radix et rhizoma] – Salvanolic acid B (Pure metabolite, purity >99%, CAS 121521-90-2, Herbpurify, Cat#115939-25-8, Lot#190322 R)	*In vivo*: 100 mg/kg/d *In vitro*: 25, 50, 100 µM	Not specified	*In vivo*: (C-BSA-induced MN rat model)*In vitro*: (LPS-induced human mesangial)	Positive: none; Negative: normal control (saline), model (vehicle)	*In vivo*: 2 weeks; *In vitro*: 24 h	Pure metabolite	Chen et al. (2022)
Jianpi Qushi Heluo Formula	*Astragalus membranaceus* (Fisch.) Bunge [Fabaceae; Astragali Radix]; *Atractylodes macrocephala* Koidz. [Asteraceae; Atractylodis Macrocephalae Rhizoma]; *Poria cocos* (Schw.) Wolf [Polyporaceae; Poria]; *Stephania tetrandra* S.Moore [Menispermaceae; Stephaniae Tetrandrae Radix]; *Perilla frutescens* (L.) Britton [Lamiaceae; Perillae Folium]; *Dioscorea nipponica* Makino [Dioscoreaceae; Dioscoreae Nipponicae Rhizoma]; *Panax ginseng* C.A.Mey. [Araliaceae; Ginseng Radix]; *Angelica sinensis* (Oliv.) Diels [Apiaceae; Angelicae Sinensis Radix]; *Nelumbo nucifera* Gaertn. [Nelumbonaceae; Nelumbinis Folium]	16.2 g crude drug/kg/day	Not specified	*In vivo* (anti-Fx1A serum-induced PHN rats)	Positive: Benazepril (1 mg/100g/day), Tetrandrine (0.5 mg/100g/day) Negative: normal control, model group (water)	4 weeks	Traditional Chinese medicine decoction	Wang et al. (2021c)
Jianpi Qushi Heluo Formula	*Astragalus membranaceus* (Fisch.) Bunge [Fabaceae; Astragali radix], *Atractylodes macrocephala* Koidz. [Asteraceae; Atractylodis macrocephalae rhizoma], *Poria cocos* (Schw.) Wolf [Polyporaceae; Poria], *Stephania tetrandra* S. Moore [Menispermaceae; Stephaniae tetrandrae radix], *Perilla frutescens* (L.) Britton [Lamiaceae; Perillae folium], *Dioscorea nipponica* Makino [Dioscoreaceae; Dioscoreae nipponicae rhizoma], *Panax ginseng* C. A. Mey. [Araliaceae; Ginseng radix et rhizoma], *Angelica sinensis* (Oliv.) Diels [Apiaceae; Angelicae sinensis radix], *Nelumbo nucifera* Gaertn. [Nelumbonaceae; Nelumbinis folium]	16.2, 32.4 g/kg/day	Not specified	*In vivo* (anti-Fx1A serum-induced PHN rats); *In vitro* (C5b-9-stimulated podocyte injury model)	Positive: benazepril (10 mg/kg/day); Negative: normal saline/untreated model	*In vivo*: 6 weeks; *In vitro*: 24 h	Aqueous extract (decoction)	Yan et al. (2025)
Madecassoside	Madecassoside (from *Centella asiatica* (L.) Urb. [Apiaceae; Centellae asiaticae herba])	30 mg/kg/day	Not specified	*In vivo* (PHN rat model)	Negative: sham/untreated model	4 weeks	Pure metabolite	Liu et al. (2025a)
Wenyang Lishui Decoction	*Aconitum carmichaelii* Debx. [Ranunculaceae; Aconiti lateralis radix praeparata], *Astragalus membranaceus* (Fisch.) Bunge [Fabaceae; Astragali radix], *Poria cocos* (Schw.) Wolf [Polyporaceae; Poria], *Atractylodes macrocephala* Koidz. [Asteraceae; Atractylodis macrocephalae rhizoma], *Paeonia lactiflora* Pall. [Paeoniaceae; Paeoniae radix alba], *Zingiber officinale* Rosc. [Zingiberaceae; Zingiberis rhizoma recens]	*In vivo*: 16.5 g/kg/day; *In vitro*: 2, 4, 8 mg/mL	Not specified	*In vivo* (C-BSA-induced MN rat model); *In vitro* (MN patient serum-induced podocyte injury model)	Positive: benazepril (10 mg/kg/day); Negative: normal saline/untreated model/healthy volunteer serum	*In vivo*: 4 weeks; *In vitro*: 24 and 48 h	Aqueous extract	Lu et al. (2020)
Sanqi oral solution	*Astragalus mongholicus* Bunge [Fabaceae; Astragali radix], *Panax notoginseng* (Burkill) F.H. Chen [Araliaceae; Notoginseng radix]	*In vivo*: 12.6 mg/kg/day; *In vitro*: 600 μg/mL	Not specified	*In vivo* (PHN rat model); *In vitro* (ADR-induced podocyte injury model)	Positive: cyclophosphamide and prednisone; Negative: normal saline/untreated model/ADR	*In vivo*: 21 days; *In vitro*: 24 h	Aqueous extract (oral solution)	Wang et al. (2021a)
Kemeng Fang	*Codonopsis pilosula* (Franch.) Nannf. [Campanulaceae; Codonopsis radix], *Astragalus membranaceus* (Fisch.) Bunge [Fabaceae; Astragali radix], *Coptis chinensis* Franch. [Ranunculaceae; Coptidis rhizoma], *Perilla frutescens* (L.) Britton [Lamiaceae; Perillae folium], *Rehmannia glutinosa* (Gaertn.) DC. [Scrophulariaceae; Rehmanniae radix praeparata], *Ligusticum chuanxiong* Hort. [Apiaceae; Chuanxiong rhizoma], *Euryale ferox* Salisb. [Nymphaeaceae; Euryales semen], *Sabia japonica* Maxim. [Sabiaceae; Sabiae caulis], *Rhus chinensis* Mill. [Anacardiaceae; Rhois chinensis folium], *Lobelia chinensis* Lour. [Campanulaceae; Lobeliae chinensis herba], *Oldenlandia diffusa* (Willd.) Roxb. [Rubiaceae; Oldenlandiae diffusae herba]	Low: 3.3075 g/kg/day; Medium: 6.615 g/kg/day; High: 13.23 g/kg/day	Not specified	*In vivo* (CBSA-induced MN rat model)	Positive: benazepril (9 mg/kg/day), PI3K inhibitor LY294002; Negative: normal saline/untreated model	4 weeks	Aqueous extract (decoction)	Li et al. (2024)
Tetrandrine	Tetrandrine (C_38_H_42_N_2_O_6_, HPLC≥98%), a bisbenzylisoquinoline alkaloid isolated from *Stephania tetrandra* S.Moore [Menispermaceae; Stephaniae Tetrandrae Radix]	20 mg/kg/day	Not specified	*In vivo* (Fx1A antiserum-induced Heymann Nephritis rat model)	Positive: None Negative: normal control, HN model group (distilled water)	4 weeks	Pure metabolite	Yin et al. (2021)
Dhydroartemisinin	*Artemisia annua* L. [Asteraceae; Artemisiae annuae herba] – Dihydroartemisinin (purity not specified)	*In vivo*: 10 μM	Not specified	*In vivo*: (C-BSA-induced MN mouse model)	Positive: none; Negative: normal control, model	3 weeks	Pure metabolite	Li et al. (2023b)
Moshen granule	*Astragalus membranaceus* (Fisch.) Bunge [Fabaceae; Astragali radix], *Atractylodes macrocephala* Koidz. [Asteraceae; Atractylodis macrocephalae rhizoma], *Eucommia ulmoides* Oliv. [Eucommiaceae; Eucommiae cortex], *Plantago asiatica* L. [Plantaginaceae; Plantaginis semen], *Lycium chinense* Mill. [Solanaceae; Lycii fructus], *Cryptotympana pustulata* Walker [Cicadidae; Cicadae periostracum], *Euonymus alatus* (Thunb.) Siebold [Celastraceae; Euonymi alati ramulus], *Sinomenium acutum* (Thunb.) Rehder and E.H.Wilson [Menispermaceae; Sinomenii caulis], *Dioscorea nipponica* Makino [Dioscoreaceae; Dioscoreae nipponicae rhizoma], *Patrinia scabiosifolia* Link [Caprifoliaceae; Patriniae herba], *Rosa laevigata* Michx. [Rosaceae; Rosae laevigatae fructus], *Schisandra chinensis* (Turcz.) Baill. [Schisandraceae; Schisandrae chinensis fructus], *Eupolyphaga sinensis* Walker [Polyphagidae; Eupolyphaga], *Angelica sinensis* (Oliv.) Diels [Apiaceae; Angelicae sinensis radix]	1.85, 3.70 g/kg/day	Not specified	*In vivo* (CBSA-induced MN rat model)	Negative: normal saline/untreated model	4 weeks	Aqueous extract (granule)	Wang et al. (2023c)
Astragaloside IV	Astragaloside IV from *Astragalus membranaceus* (Fisch.) Bunge [Fabaceae; Astragali radix]	*In vitro*: 10, 20, 40 μM	Not specified	*In vitro* (ZAS-Induced Podocyte Injury Model)	Negative: untreated model/ZAS	24 h	Pure metabolite	Wang et al. (2023c)
Rhodojaponin VI	Rhodojaponin VI, a grayanane diterpenoid isolated from *Rhododendron molle* G. Don [Ericaceae; Rhododendri Mollis Radix]	0.02 mg/kg/day	Not specified	*In vivo* (PHN rat model); *In vitro*: (C5b-9-induced human podocyte model)	Positive: FK506 (1 mg/kg/day) Negative: sham control, PHN model group (distilled water)	4 weeks	Pure metabolite	Song et al. (2024)
Sanqi Qushi formula	*Astragalus mongholicus* Bunge [Fabaceae; Astragali radix], *Panax notoginseng* (Burkill) F.H. Chen [Araliaceae; Notoginseng radix], *Curcuma phaeocaulis* Valeton [Zingiberaceae; Curcumae rhizoma], *Paeonia lactiflora* Pall. [Ranunculaceae; Paeoniae radix rubra], *Atractylodes macrocephala* Koidz. [Asteraceae; Atractylodis macrocephalae rhizoma], *Smilax glabra* Roxb. [Smilacaceae; Smilacis glabrae rhizoma], *Isaria cicadae* Miquel [Cordycipitaceae; Chanhua]	12.6, 25.2 g/kg/day	Not specified	*In vivo* (PHN rat model)	Positive: cyclophosphamide (15 mg/kg/day); Negative: normal saline/untreated model	3 weeks	Aqueous extract (decoction)	Lin et al. (2025)
Sanqi Qushi formula	*Panax notoginseng* (Burkill) F.H.Chen [Araliaceae; Notoginseng radix], *Astragalus membranaceus* (Fisch.) Bunge [Fabaceae; Astragali radix], *Smilax glabra* Roxb. [Smilacaceae; Smilacis glabrae rhizoma], *Atractylodes macrocephala* Koidz. [Asteraceae; Atractylodis macrocephalae rhizoma], *Cordyceps cicadae* (Miq.) Massee [Cordycipitaceae; Cordyceps cicadae], *Curcuma phaeocalis* Valeton [Zingiberaceae; Curcumae rhizoma], *Paeonia lactiflora* Pall. [Paeoniaceae; Paeoniae radix rubra]	5.3, 10.6, 21.2 g/kg/day	Not specified	*In vivo* (PHN rat model); *In* *vitro* (ZAS-Induced Injury immortalized mouse podocytes)	Positive: tacrolimus (0.5 mg/kg/day), 4-PBA; Negative: normal saline/untreated model/ZAS	*In vivo*: 21 days; *In* *vitro*: 24 h	Aqueous extract (decoction)/medicated serum	Sun et al. (2025)
Sanqi oral solution	*Astragalus mongholicus* Bunge [Fabaceae; Astragali radix], *Panax notoginseng* (Burkill) F.H. Chen [Araliaceae; Notoginseng radix]	*In vivo*: 6.3, 12.6 mL/kg/day *In vitro*: 600 μg/mL	Not specified	*In vivo* (PHN rat model) *In vitro* (ADR-induced podocyte injury model)	Positive: tacrolimus (0.315 mg/kg/day) Negative: normal saline/untreated model/ADR	*In vivo*: 21 days *In vitro*: 24 h	Aqueous extract (oral solution)	Wang et al. (2025b)
Jianpi Qushi Heluo Formula	*Astragalus membranaceus* (Fisch.) Bunge [Fabaceae; Astragali radix], *Atractylodes macrocephala* Koidz. [Asteraceae; Atractylodis macrocephalae rhizoma], *Poria cocos* (Schw.) Wolf [Polyporaceae; Poria], *Stephania tetrandra* S. Moore [Menispermaceae; Stephaniae tetrandrae radix], *Perilla frutescens* (L.) Britton [Lamiaceae; Perillae folium], *Dioscorea nipponica* Makino [Dioscoreaceae; Dioscoreae nipponicae rhizoma], *Panax ginseng* C. A. Mey. [Araliaceae; Ginseng radix et rhizoma], *Angelica sinensis* (Oliv.) Diels [Apiaceae; Angelicae sinensis radix], *Nelumbo nucifera* Gaertn. [Nelumbonaceae; Nelumbinis folium]	16.2, 32.4 g/kg/day	≥16.2 g/kg	*In vivo* (anti-Fx1A antiserum induces the PHN rat model)	Positive: benazepril (10 mg/kg/day); Negative: normal saline/untreated model	4 weeks	Aqueous extract (decoction)	Wang et al. (2022b)
Moshen Fuyuan Formula	*Astragalus membranaceus* Bunge [Fabaceae; Astragali radix], *Salvia miltiorrhiza* Bunge [Lamiaceae; Salviae miltiorrhizae radix], *Atractylodes macrocephala* Koidz. [Asteraceae; Atractylodis macrocephalae rhizoma], *Stephania tetrandra* S. Moore [Menispermaceae; Stephaniae tetrandrae radix], *Poria cocos* (Schw.) Wolf [Polyporaceae; Poria], *Coix lacryma-jobi* L. [Poaceae; Coicis semen], *Curcuma aromatica* Salisb. [Zingiberaceae; Curcumae radix], *Reynoutria japonica* Houtt. [Polygonaceae; Polygoni cuspidati rhizoma], *Lonicera japonica* Thunb. [Caprifoliaceae; Lonicerae japonicae flos], *Dioscorea nipponica* Makino [Dioscoreaceae; Dioscoreae nipponicae rhizoma]	Not specified	Not specified	MN patients	Healthy controls (for microbiome); pre-treatment as baseline	12 weeks	Aqueous extract (granule)	Wang et al. (2024b)

## 3 Clinical effectiveness of CHMs in treating MN

MN is caused by multiple predisposing factors such as immune abnormalities, genetics, infections, and environmental factors. Although significant progress has been made in understanding the pathogenesis of MN, corresponding treatments remain lagging, severely impacting patients’ quality of life and posing a prominent medical challenge and public health issue. MN is recognized in TCM as “urinary turbidity,” “edema,” and “consumptive disease,” with a millennia-long history of application in treating such conditions (Lang et al., 2020), yielding favorable therapeutic outcomes. Recent clinical studies have also validated the efficacy of botanical drug formulations such as MFSD, Tripterygium wilfordii polyglycoside tablets, Kunxian capsule, Shulifenxiao Formula, Wuzhi capsules, JQHF, and CRT (Dong et al., 2021; Guo et al., 2021; Gao et al., 2021; Zhang et al., 2024b; Lv et al., 2025; Cui et al., 2021; Zhang et al., 2019; Du et al., 2025; Wang et al., 2021c; Liu et al., 2025b). They have demonstrated positive effects in improving core MN indicators, such as reducing proteinuria, elevating serum albumin levels, and achieving clinical remission rates. Overall safety remains manageable, with adverse reactions predominantly mild and reversible. This preliminary evidence offers diverse treatment options for MN, particularly demonstrating unique therapeutic value in patients with poor response to conventional therapies or those with contraindications.

### 3.1 Randomized controlled clinical trials of MN treatment using CHMs

A study conducted by the Nephrology Department of the 32298th Hospital of the Chinese People’s Liberation Army aimed to evaluate the efficacy of combining Nephritis Rehabilitation Tablets with tacrolimus in treating IMN. A total of 84 high-risk IMN patients (defined as 24-h UPE ≥4 g/d and pathological stage I-II) were enrolled (Lv et al., 2021). These patients were randomly divided into the study group (42 cases) and the control group (42 cases). All patients received conventional symptomatic treatments such as glucocorticoids, antihypertensives, lipid-lowering, anticoagulants, and diuretic detumescence, as well as tacrolimus treatment (0.05–0.1 mg/(kg·d), oral administration, BID). The study group was additionally administered Nephritis Rehabilitation Tablets (1.5 g per dose, TID). The treatment course was 12 weeks. The results showed that the total effective rate of the study group was significantly higher than that of the control group; SCr and 24h-UPE were both lower than those of the control group, while ALB was higher; the levels of IgG4 and C5b-9 were significantly decreased; and there was no statistically significant difference in the incidence of adverse reactions.

To validate the efficacy and safety of TWM therapy for low-to-intermediate-risk IMN, Sichuan Provincial People’s Hospital designed and registered a prospective, single-center, open-label randomized controlled clinical trial (RCT) (Geng et al., 2022). The study plan enrolled 20 patients with IMN confirmed by renal biopsy and 24h-UPE of 1.0–3.5 g. Participants were randomized in a 1:1 ratio to either the intervention group (TWG 1–1.5 mg/kg/day combined with ACE inhibitor/ARB) or the control group (ACE inhibitor/ARB), with a treatment cycle of 6 months. The primary endpoint was the absolute reduction in proteinuria post-treatment. Secondary endpoints included the reduction in urine albumin-creatinine ratio, clinical remission rate (complete remission: proteinuria <0.3 g/day and albumin ≥3.5 g/dL; partial remission: ≥50% reduction in proteinuria), and safety metrics such as hepatotoxicity and reproductive toxicity. The study protocol employed sealed envelope randomization, adjusted baseline proteinuria differences using covariance analysis, and enhanced safety monitoring through high-frequency follow-ups. Limitations include a small sample size and a single-center design, which may restrict the generalizability of findings. Future multicenter validation with expanded samples is needed to optimize treatment strategies for low-to-intermediate-risk IMN patients.

In a multicenter, randomized, double-blind, controlled trial (ChiCTR2200061953) jointly conducted by five TCM hospitals in China, 130 adult patients with high-risk IMN (proteinuria >3.5 g/d and ALB <25 g/L or anti-PLA2R >50 RU/mL) were enrolled. The patients were randomly divided into two groups: the treatment group receiving SQG (1 sachet per dose, TID) combined with cyclophosphamide pulse therapy plus glucocorticoid therapy, and the control group receiving placebo combined with the same basic regimen. All patients received basic treatments such as antihypertensive therapy with ACEI/ARB. The intervention lasted for 6 months, followed by a 6-month follow-up. Results showed that the SQG group exhibited significantly higher TR at 6 months of treatment compared to the control group. Concurrently, sustained improvement trends were observed in 24h-UPE quantification, anti-PLA2R antibody titers, and TCM dampness syndrome scores. By the end of follow-up, the SQG group maintained a significant remission advantage without reporting any drug-related serious adverse events (Li et al., 2023a). This indicates that the combination regimen can enhance CR and PR rates while reducing the risk of immunosuppressive agent side effects.

### 3.2 Systematic reviews and meta-analyses of treating MN with CHMs

Astragalus membranaceus is rich in a variety of characteristic bioactive metabolites, such as calycosin, formononetin, etc., (Xu et al., 2024a), and is hailed as a qi-tonifying medicinal botanical drug in TCM, serving as a core component of various TCM compound prescriptions. A meta-analysis encompassing 50 studies and 3,423 participants specifically evaluated the efficacy and safety of Astragalus-based formulas in treating moderate-to-high-risk IMN. Results showed that Astragalus preparations combined with conventional therapy (supportive care or immunosuppressive therapy) significantly improved CR [RR = 1.63, 95% CI (1.46, 1.81), P < 0.001] and PR rates [RR = 1.13, 95% CI (1.05, 1.20), P = 0.0004] compared with conventional therapy alone. It also reduced 24h-UPE [MD = −1.05 g/24h, 95% CI (−1.21, −0.89), P < 0.001], SCr [MD = −6.24 μmol/L, 95% CI (−9.85, −2.63), P = 0.0007], and increased ALB [MD = 3.75, 95% CI (3.01, 4.49), P < 0.001]. The treatment group demonstrated superior efficacy compared to the control group without a significant increase in the risk of adverse reactions (Wang et al., 2023a). Additionally, the efficacy of self-formulated Chinese botanical drug combinations containing Astragalus in IMN treatment has been validated. The effectiveness and safety of the botanical drug formulas Self-Developed Qi Xue Shui Mo Shen Prescription and Self-Developed Ye Shi Yi Shen Xiao Bai Formula when used in combination with conventional therapy for IMN were evaluated in a meta-analysis. This analysis involved 126 Chinese subjects across two studies. Results demonstrated that the Chinese botanical compound (Self-Developed Ye Shi Yi Shen Xiao Bai Formula) combined with conventional treatment significantly reduced 24-h urinary protein excretion [MD = −3.16 g/24h, 95% CI (−4.03, −2.29)]. Although the pooled analysis of two studies showed MD = −1.57 g/24h, it exhibited high heterogeneity. Regarding TR, no significant difference was observed between combination therapy and conventional therapy [RR = 1.09, 95% CI (0.92, 1.29)]. Regarding renal function, improvements in SCr and BUN levels in the treatment group were comparable to those in the control group. Similarly, improvements in TC, TG, and recurrence rates showed no significant difference between the two groups (Liu et al., 2021).

TWM, as a commonly used Chinese botanical drug extract, has demonstrated therapeutic efficacy in MN treatment, supported by multiple systematic reviews. A study aimed to analyze the efficacy and safety of TWM in the treatment of MN. The study included 30 RCTs involving 13 intervention measures, with a total of 2410 IMN patients. The control group was treated with GC, CNI, and other therapies, while the experimental group received a monotherapy or combination regimen containing TWM. The results showed that the treatment regimen containing TWM was superior to the traditional GC and CNI regimens in terms of efficacy, with no significant difference in safety (Wang et al., 2023b). Another systematic review and meta-analysis on the efficacy and safety of TWM for treating glomerulonephritis included 16 studies, comprising 6 randomized controlled trials and 10 cohort studies involving 1,065 participants. These studies addressed MN, DKD, and HSPN, with the control group receiving conventional therapy (GC, valsartan, etc.) and the experimental group receiving combination regimens containing TWM. Results demonstrated that TWM combination therapy improved remission rates, reduced 24h-UPE, and decreased recurrence. Its efficacy was comparable to that of GC and valsartan, with no significant increase in adverse reactions such as infections or liver damage (Yan et al., 2024b). However, both studies have limitations. The former includes the following: inconsistent follow-up times and drug dosages among the included studies, which may affect the reliability of the results; the potential spontaneous remission of IMN may interfere with the judgment of TR; some intervention measures have a small number of studies and small sample sizes, which may lead to false positive results; the included studies are mainly from China, resulting in geographic bias; there may be differences in treatment responses among different age groups; long-term efficacy data are lacking, such as recurrence rate and survival rate; some RCTs did not clearly report details such as randomization and allocation concealment, which may lead to bias; there is insufficient data on anti-PLA2R antibodies to conduct relevant analyses; there are more studies on medium and high-risk patients and fewer on low-risk patients, making it difficult to distinguish the efficacy among patients with different risk levels. The latter includes a limited number of included studies and cases, making it difficult to conduct subgroup analysis, sensitivity analysis, and assess publication bias, with significant heterogeneity among studies. Therefore, the long-term efficacy and suitable population of TWM treatments still need to be further clarified.

In 2024, Zhu et al. found in a meta-analysis that included 31 randomized controlled trials involving 2195 patients and systematically evaluated the efficacy of the combination therapy of 15 kinds of Chinese botanical drugs including Wuzhi Capsules, Tripterygium Glycosides Tablets (LGT), and Huangkui Capsules with Biomedicine (BM) that the combination therapy was superior to pure BM treatment in improving various renal disease indicators. Moreover, different CHMs had respective prominent advantages in reducing urinary protein, creatinine, blood lipids, and side effects, with all improvements being statistically significant (P < 0.05) (Zhu et al., 2024). Similarly, in 2021, a systematic review and meta-analysis by Lu et al. also supported the advantages of combination therapy. This study encompassed 29 randomized controlled trials involving 1,883 participants, evaluating the efficacy of integrating TCM therapies—such as SQG, Qingre Huoxue Yishen Decoction, and TWM—with conventional Western medicine. It found that the total effective rate (OR = 2.59, P = 0.003) and cure rate (OR = 3.01, P = 0.01) of TCM combined with basic treatment were superior to basic treatment alone. The overall response rate of TCM combined with immunosuppressants (OR = 3.01, P = 0.01) was also superior to basic treatment alone. P = 0.003) and cure rate (OR = 3.01, P = 0.01) were superior to basic therapy alone. The total effective rate (OR = 3.01, P < 0.00001) and cure rate (OR = 1.73, P = 0.02) of TCM combined with immunosuppressants were also significantly higher than those of immunosuppressant monotherapy. Furthermore, combination therapy reduced recurrence rates (OR = 0.28, P = 0.0004) and adverse event incidence (OR = 0.38, P < 0.00001), demonstrating particularly pronounced effects in decreasing pulmonary infections and Cushing’s syndrome (Lu et al., 2021). Overall, both studies indicate that combining TCM with Western medicine for treating IMN yields superior outcomes compared to Western medicine alone in terms of enhancing efficacy and reducing adverse reactions. Different CHMs also demonstrate distinct characteristics in improving specific indicators. However, both studies share methodological limitations, such as inclusion of studies with suboptimal quality, inadequate description of randomization methods, lack of allocation concealment and adequate blinding, small sample sizes, and a predominance of single-center studies. Therefore, future research should rely on multicenter, large-sample, rigorously designed randomized controlled trials to further clarify the optimal combination therapy regimen for IMN and validate its long-term efficacy and safety.

### 3.3 Safety and interactions of combination therapies

A critical appraisal of the safety profile of CHMs, particularly when combined with conventional immunosuppressive or supportive therapies, is essential for clinical translation. Analysis of included clinical studies reveals both the promise and complexity of managing safety in integrative regimens. Understanding the underlying mechanisms of botanical drug-drug interactions and toxicity is key to safe clinical practice.

Several CHMs derived from Tripterygium wilfordii, such as Tripterygium wilfordii polyglycoside tablets, exhibit potent efficacy but carry well-documented toxicity in combination regimens. The primary active metabolite, triptolide, exerts immunosuppressive effects by inhibiting lymphocyte proliferation and NF-κB activation (Guo et al., 2021; Liu et al., 2015). However, triptolide is also responsible for its narrow therapeutic window. Mechanistically, triptolide-induced hepatotoxicity involves excessive oxidative stress and mitochondrial dysfunction, leading to apoptosis via the mitochondrial pathway. In 2024, Hu et al. demonstrated that triptolide-induced liver injury is linked to modulation of the bile acid-FXR axis (Hu et al., 2024b). Reproductive toxicity, presenting as irregular menstruation in women and oligospermia in men, arises from direct pro-apoptotic effects on germ cells and disruption of the hypothalamic–pituitary–gonadal axis. This dose-dependent toxicity necessitates regular monitoring of liver function, menstrual status, and fertility parameters. In a 2021 retrospective study of 55 primary MN patients receiving TWG combined with ARB, Guo et al. reported liver dysfunction (8.6%) and irregular menstruation (5.8%), both reversible upon treatment adjustment (Guo et al., 2021). Similarly, in 2021, Gao et al. observed irregular menstruation in one patient receiving Tripterygium wilfordii polyglycoside tablets combined with calcineurin inhibitors, which improved after the administration of traditional Chinese medicine, with no significant difference in overall adverse effects compared to the calcineurin inhibitor plus glucocorticoid group (Gao et al., 2021). Pharmacodynamic interactions between CHMs and immunosuppressants can notably enhance therapeutic efficacy and mitigate potential drug toxicities. The addition of Nephritis Rehabilitation Tablets to a tacrolimus-based therapeutic regimen for MN did not result in an increased incidence of adverse reactions. In 2021, Lv et al. conducted a randomized controlled clinical study involving 84 patients with MN (pathological stage I or II), who were equally divided into a study group and a control group. The results showed that this combination therapy significantly improved the clinical therapeutic effect, with the total effective rate reaching 90.48% in the combination group compared with 71.43% in the tacrolimus monotherapy group (P = 0.026). Meanwhile, there was no statistically significant difference in the incidence of adverse events between the two groups, which were 11.90% and 7.14%, respectively (P = 0.710). This combination regimen was proven to effectively improve renal function indices, downregulate the levels of urinary IgG4 and C5b-9, and elevate the overall therapeutic effect without increasing the risk of adverse reactions (Lv et al., 2021). This formula contains *S. miltiorrhiza* Bunge [Lamiaceae; Salviae Miltiorrhizae Radix et Rhizoma] and *A. membranaceus* (Fisch.) Bge. [Fabaceae; Astragali radix], which exert anti-inflammatory and antioxidant effects that may counteract endothelial dysfunction and oxidative stress related to calcineurin inhibitors, thereby improving renal function without added toxicity.

The Shuli Fenxiao Formula combined with low-dose cyclophosphamide has been explored as a glucocorticoid-sparing regimen. In 2025, Du et al. reported that among 31 intermediate-to-high-risk PMN patients treated for 24 weeks, adverse events included three infections and two cases of reversible liver impairment (Du et al., 2025). The steroid-free regimen achieved a 64.5% remission rate at 24 weeks while reducing glucocorticoid-related adverse effects. Importantly, SLFX monotherapy in 31 patients with refractory MN resulted in 80.7% remission at 12 months and 90.9% at 24 months, with only two relapses and no severe adverse events, as demonstrated by Cui et al. (2021), supporting its value as a steroid-sparing agent (Cui et al., 2021).

The QingreHuoxue Formula further illustrates mechanism-based combination therapy. In a C-BSA-induced MN mouse model conducted in 2024, Lou et al. found that this formula, combined with prednisolone acetate and cyclosporine A, achieved superior outcomes compared to Western medicine alone, while reducing side effects and drug dependency (Lou et al., 2024). The combination reduced Th17 cell counts and IL-17 expression and increased Treg populations, suggesting restoration of Th17/Treg balance, a key pathogenic factor in MN, which may allow lower immunosuppressant doses and reduce dose-dependent toxicities.

Despite these advances, important mechanistic gaps remain. First, the precise molecular targets of many CHM metabolites remain unclear. Although TWG reduces anti-PLA2R titers as reported by Guo et al. (2021), whether this occurs via direct B-cell suppression, reduced autoantibody production, or podocyte protection remains undefined. Second, the relative contributions of individual metabolites versus synergistic multi-constituent effects are poorly characterized. For example, Moshen granule contains 81 identified metabolites; in 2023, Wang et al. documented these metabolites (Wang et al., 2023c), and attributing its effects on the Wnt/β-catenin/RAS axis solely to astragaloside IV may oversimplify its polypharmacological actions. Future studies should prioritize target identification using affinity chromatography and cellular thermal shift assays, perform dedicated PK/PD studies to characterize botanical drug-drug interactions, and employ systems biology approaches to define multi-target effects of complex formulas.

In conclusion, the safety and toxicity profiles of CHMs in the treatment of MN are mediated by multiple identifiable mechanisms, including enzyme inhibition, direct cellular toxicity, and other related pathways. Although current clinical evidence suggests that many combination regimens are well-tolerated, ensuring patient safety relies on a thorough understanding of these underlying mechanisms. Such insight is critical not only for predicting potential botanical drug–drug interactions but also for facilitating personalized therapy and promoting safer clinical practice in integrative medicine.

## 4 Discussion

This paper reviews the pathogenesis of MN and elucidates the mechanisms of action and clinical efficacy of CHMs and their active metabolites in treating MN. CHMs exert multifaceted effects through regulating multiple signaling pathways—including NF-κB and JAK-STAT—and the complement system. These effects encompass anti-inflammatory, immunomodulatory, antioxidant, autophagy and apoptosis regulation, anti-fibrotic, and microbiota modulation functions. This demonstrates the pharmacological basis for CHMs’ “multi-target-multi-pathway” synergistic intervention in the complex pathological network of MN. This multi-step intervention characteristic makes it more adaptable to the complex pathological network of MN than single-target drugs. For example, the different active metabolites of *C. sativus* L [Iridaceae; Croci stigma] based on SIRT1-mediated multi-target intervention synergistically act on two core pathological processes, namely oxidative damage and inflammatory cascade reaction. For another example, in 2023, Ma, Wang, et al. investigated the mechanism of action of MSG on MN in C-BSA-induced MN rat models. Interestingly, these two studies found that MSG exerts its effects by respectively inhibiting AHR signaling to regulate the NF-κB/Nrf2 signaling pathway and inhibiting the Wnt1/β-catenin pathway. This is not a contradictory phenomenon, but rather precisely reflects the unique advantages of TCM treatment, such as multi-pathway, multi-target, and regulation of the body’s dynamic balance. Therefore, future research may explore the precise correspondence between “TCM syndrome patterns, molecular phenotypes, and botanical interventions,” advancing MN therapy from “single-target” to “systemic regulation.”

Through a review of the mechanisms underlying MN, this paper identifies the following limitations in basic research on MN. With regard to experimental models, existing preclinical models of MN fail to encompass all clinical triggers. Most studies reviewed herein are based on either the PHN model or the C-BSA-induced rat MN model. However, a fundamental limitation persists: these models do not fully recapitulate human pathophysiology, particularly the autoimmune response mediated by anti- PLA2R antibodies, which represent the hallmark of idiopathic MN in humans. For instance, the PHN model targets the megalin antigen (Ronco and Debiec, 2010), which is not expressed in human podocytes, whereas the C-BSA model relies on immune complex deposition without involving podocyte-specific antigens. Accordingly, mechanistic insights derived from these models—especially those related to antigen-specific immune regulation—should be interpreted with caution. Future research should prioritize the development of PLA2R-based transgenic or humanized animal models to better mimic human disease and enhance the translational relevance of preclinical findings.

In addition to the limitations inherent in animal models, the relevance and interpretability of *in vitro* models employed in MN research warrant critical examination. *In vitro* systems—such as TNF-α-stimulated MPC5 podocytes, C5b-9-induced podocyte injury models, or ADR-triggered apoptotic assays—provide valuable platforms for dissecting molecular pathways and evaluating the direct cytoprotective, anti-inflammatory, or antioxidant effects of CHMs constituents under highly controlled conditions. They enable high-throughput screening and mechanistic investigations that are often impractical *in vivo*. However, several fundamental limitations must be considered when extrapolating *in vitro* findings to the complex pathophysiology of MN. First, the concentrations of active metabolites applied *in vitro* frequently exceed the plasma or tissue levels achievable after oral administration of CHMs, raising concerns about pharmacological relevance. Second, immortalized cell lines such as MPC5, although widely used, may exhibit phenotypic alterations compared to primary podocytes and lack the intricate interactions with other renal cell types that characterize the glomerular microenvironment in MN. Third, *in vitro* models cannot recapitulate systemic factors critical to MN pathogenesis, including humoral immune responses, complement cascade activation, and the dynamic crosstalk between the kidney and the immune system. Consequently, while *in vitro* experiments can demonstrate that a given CHM possesses anti-inflammatory or anti-apoptotic properties, such observations alone do not prove therapeutic efficacy in the multifaceted *in vivo* context of MN. They should be regarded as hypothesis-generating tools that require validation in appropriate animal models and, ultimately, in clinical studies. Future research should aim to integrate pharmacokinetic–pharmacodynamic analyses to bridge the gap between *in vitro* concentrations and *in vivo* exposures, and to employ more physiologically relevant systems—such as co-cultures, kidney organoids, or precision-cut kidney slices—that better mimic the disease milieu.

Another important consideration is the translatability of effective doses observed in animal studies to clinical application in humans. For instance, metabolites such as diosgenin (10 mg/kg), safranal (200 mg/kg), and crocin (100 mg/kg) have shown renal protective effects in rodent models of MN. However, direct extrapolation of these doses to humans is not straightforward due to interspecies differences in metabolism, bioavailability, and body surface area. According to standard dose conversion guidelines based on body surface area, the equivalent human dose for a rat dose of 10 mg/kg would be approximately 1.6 mg/kg. Even so, such conversions do not account for potential differences in drug absorption, distribution, and target engagement. Therefore, while animal studies provide valuable mechanistic insights, further pharmacokinetic and dose-finding studies in humans are essential to establish safe and effective dosing regimens for clinical application.

While it is encouraging that the majority of reviewed studies employed multiple dose groups to explore dose-dependent effects—a methodological strength reflected in Table 2—several critical gaps remain in the dose-response characterization of CHMs in MN. First, although multi-dose designs were used, the minimal effective dose or concentration was rarely explicitly identified or statistically validated. As indicated by the predominance of “Not specified” entries in the Minimal active concentration column of Table 2, most studies did not perform post-hoc comparisons to determine the lowest dose that significantly differed from the model control. This omission limits the translational utility of these findings, as the therapeutic window cannot be accurately defined without knowing both the minimal effective dose and the maximal tolerated dose. Second, and more fundamentally, no study to date has integrated pharmacokinetic and pharmacodynamic modeling. The current evidence base is largely phenomenological: it establishes that a given dose produces a given effect, such as reduced proteinuria. However, it does not answer the crucial question of what concentration of the active constituents in the blood or target tissue is required to achieve that effect, nor does it elucidate the temporal relationship between drug exposure and response. Without such pharmacokinetic and pharmacodynamic data, rational dose selection for clinical trials remains speculative. Future research should therefore move beyond simply demonstrating dose-dependency toward a more rigorous quantitative framework. This requires explicitly reporting and statistically validating the minimal effective dose, conducting dedicated pharmacokinetic studies to characterize the absorption and bioavailability of key marker compounds, and employing pharmacokinetic and pharmacodynamic modeling to establish concentration-response relationships. Such an approach would transform the current descriptive understanding of CHM efficacy into a predictive, mechanism-based pharmacology that can reliably guide clinical translation.

This challenge becomes even more pronounced when considering complex CHM formulas rather than isolated metabolites. Preclinical studies of single metabolites operate within a reductionist framework that assumes a direct, dose-dependent relationship between a specific metabolite and its therapeutic effect. Yet most CHMs are administered as multi-botanical drug decoctions, where the putative “active” metabolites may be present only in trace amounts. Their observed efficacy likely arises from polypharmacology—weak interactions across multiple targets—and synergistic interplay among metabolites, where one metabolite may enhance the solubility, metabolism, or receptor binding of another. In this context, the very concept of a singular “optimal dose” becomes problematic. Future research must therefore move beyond simplistic dose-finding and embrace systems-level approaches that can decipher how the collective action of a formula’s metabolites translates into therapeutic efficacy.

A critical consideration when interpreting the findings of this review is that the validity and reproducibility of research on CHMs are often constrained by fundamental issues related to the materials themselves. It is important to acknowledge that not all of the original studies cited provided detailed authentication of the botanical materials used (e.g., via DNA barcoding or voucher specimen deposition). Consequently, the taxonomic validity of some studied CHMs cannot be fully ascertained, introducing a potential source of heterogeneity. This challenge of taxonomic uncertainty, however, is symptomatic of a deeper issue: the inherent complexity and variability of CHMs, which pose significant obstacles to standardization and clinical translation. First, substantial heterogeneity exists among botanical drug formulations, including differences in species, proportions, and preparation methods. The lack of authentication measures (e.g., pharmacopoeial compliance) increases the risk of species confusion, such as between *A. membranaceus* and *A. mongholicus*. Second, chemical standardization is frequently inadequate; most studies fail to quantify marker metabolites or apply chromatographic fingerprinting, making it difficult to ensure batch-to-batch consistency. Third, dose reporting based on crude botanical weight does not reflect the actual concentrations of active metabolites reaching systemic circulation, especially given the poor bioavailability and extensive first-pass metabolism of many compounds. Together, these limitations fundamentally challenge the reproducibility and clinical translation of the findings.

To address these limitations, future research should prioritize the use of chemically standardized extracts with quantified active constituents and incorporate pharmacokinetic studies to guide dose selection. Adherence to established reporting guidelines, such as ConPhyMP, is essential to enhance transparency and reproducibility. Furthermore, systems pharmacology approaches may help elucidate the multi-target mechanisms of CHMs and support their rational integration with conventional therapies. Ultimately, well-designed, multicenter randomized controlled trials are needed to validate preclinical findings and facilitate the evidence-based translation of CHMs into clinical practice.

This review highlights the multi-target mechanisms of CHMs in MN, with frequent references to the modulation of pathways such as NF-κB. However, the significance of these findings must be critically evaluated in the context of drug development. The demonstration of NF-κB inhibition, while common, has yet to yield a clinically approved drug for MN. This discrepancy points to several key limitations in the current body of evidence. Mechanistic studies are often descriptive in nature. Although changes in phosphorylated protein levels are routinely reported, rigorous loss- or gain-of-function experiments are rarely performed to confirm that modulation of a specific target is necessary for the observed therapeutic effect of a CHM.To advance the field, future research must move beyond phenomenological observations toward hypothesis-driven mechanistic validation. Specifically, studies should employ modern target identification technologies—such as cellular thermal shift assays and drug affinity responsive target stability analysis—to pinpoint the direct molecular targets of CHM constituents. These approaches should be integrated with genetic tools, including CRISPR/Cas9-mediated knockout or knockdown, to establish causality between target engagement and therapeutic efficacy.

It is worth noting that the occurrence and development of MN are closely linked to genetic factors (Gu et al., 2021), but research on TCM in this field remains virtually non-existent. This undoubtedly represents a critical gap that needs to be addressed in the study of TCM treatment mechanisms. In the modernization of TCM, the precise identification of active metabolites remains a core challenge. Although basic research has demonstrated the efficacy of PR-CSEE against MN, the specific metabolites responsible for its primary effects have not been thoroughly investigated. Modern studies confirm that multiple active metabolites in TCM—including notoginsenosides, AS-IV, and tripteryl lactones—exhibit ideal pharmacological properties for treating MN. However, research has primarily focused on *in vitro* experiments and animal studies, with a severe lack of clinical trial data.

Regarding clinical efficacy, this paper evaluated relevant clinical research evidence, including clinical efficacy studies, RCTs, systematic reviews, and meta-analyses. It confirmed the potential of TCM formulas or combination regimens in reducing proteinuria and improving remission rates. However, in the process of the clinical application and promotion of TCM, a series of practical problems are gradually emerging and progressively restricting the quality of its development. First, the number of RCTs on TCM treatment for MN remains limited, and most studies have been published in Chinese-language journals. This paper exclusively selected and reviewed RCTs published in English-language journals to ensure the international comparability and methodological rigor of the research findings. Results that TCM holds potential in improving renal function, reducing proteinuria, and increasing remission rates. However, limitations exist, including small sample sizes, a high proportion of single-center studies, short follow-up periods, restricted population coverage, and limited comparative protocols. Future research should therefore expand sample sizes, conduct multicenter studies, refine patient stratification, extend follow-up durations, and clarify the appropriate populations and superior efficacy of different TCM regimens. This will provide more robust evidence to support clinical treatment decisions. Secondly, the full release of the clinical value of TCM is still confronted with the bottleneck of interpreting scientific mechanisms. Owing to its complex therapeutic characteristics of multi-metabolite synergy and multi-target action, many of its curative effects still cannot be fully explained by modern pharmacology theories to date. The lag in such mechanism research has resulted in the lack of solid scientific theoretical support for the clinical application of TCM, and has also become a fundamental obstacle restricting its long-term development and in-depth promotion.

## Nomenclature

ADAM10: A Disintegrin And Metalloproteinase 10
AHR: aryl hydrocarbon receptor
AKT: protein kinase B
ALB: serum albumen
ASC: apoptosis-associated speck-like protein containing a CARD
BAD: bcl-2 agonist of cell death
Bax: BCL-2-associated X protein
Bcl-2: B cell lymphoma-2
BMSCs: bone marrow mesenchymal stem cells
BNIP3: Bcl-2/adenovirus E1B 19 kDa-interacting protein 3
BUN: blood urea nitrogen
C3: complement component 3
CAT: catalase
C-BSA: cationized bovine serum albumin
CD20: cluster of differentiation 20
CHMs: Traditional Chinese Botanical medicines
CIC: circulating immune complexes
CNI: Calcineurin inhibitors
COX-2: cyclooxygenase-2
CR: Complete Remission
Cyt c: cytochrome c
eGFR: estimated glomerular filtration rate
EMT: epithelial - mesenchymal transition
eNOS: Endothelial nitric oxide synthase
ERK: extracellular signal-regulated kinase
ESKD: end-stage kidney disease
FGF2: Fibroblast Growth Factor 2
FX1A: fraction 1A
GBM: glomerular basement membrane
GC: Glucocorticoids
GCLC: glutamate-cysteine ligase catalytic subunit
GCLM: glutamate-cysteine ligase modulatory subunit
GSH: Glutathione
HO-1: heme oxygenase-1
HSPN: henoch-schönlein purpura nephritis in children
ICAM-1: intercellular adhesion molecule-1
IFN-γ: Interferon-γ
IgG: immunoglobulin G
IKKβ: inhibitor of κB kinase β
IL-1β: Interleukin-1β
IL-6: Interleukin-6
iNOS: inducible nitric oxide synthase
IκB: inhibitor of NF-κB
IκBα: inhibitor of κBα
JAK: Janus kinase
K48: lysine 48
Keap1: Kelch-like ECH-associated protein 1
LC3-II: microtubule-associated protein 1 light chain 3-II
LDL: low-density lipoprotein
MAPK: mitogen-activated protein kinases
MCP-1: monocyte chemotactic protein-1
MDA: malondialdehyde
MDM2: Mouse Dermoid Microsomes 2
MEK: Mitogen-activated protein kinase kinase
MIR: myocardial ischemia-reperfusion
MMP-7: matrix metalloproteinase-7
MN: membranous nephropathy
MnSOD: manganese superoxide dismutase
MPC-5: mouse podocyte clone-5
NLRP3: NOD-like receptor pyrin domain-containing protein 3
NQO1: Nadhiquinone Oxidoreductase 1
Nrf2: nuclear factor erythroid 2-related factor 2
p38: The p38 group of MAP kinases
Parkin: parkin RBR E3 ubiquitin-protein ligase
PHN: passive Heymann nephritis
PI3K: phosphatidylinositol 3-kinase
PINK1: PTEN-induced kinase 1
PLA2R: phospholipase A2 receptor
PLA2R: M-type phospholipase A2 receptor
PR: Partial Remission
RAS: renin-angiotensin system
ROS: reactive oxygen species
SCr: serum creatinine
Sirt1: sirtuin 1
SMAD: the Smad transcription factors
SOD: Superoxide Dismutase
sPLA2-IB: secretory phospholipase A2 group IB
STAT: signal transducer and activator of transcription
T-bet: T-box is expressed in T cells.
TC: total cholesterol
TCM: Traditional Chinese Medicine
TG: Triglycerides
TGF-β: transforming growth factor ß
THSD7A: thrombospondin type 1 domain-containing 7A
TLR-4: toll-like receptor 4
TNF-α: tumour necrosis factor α
TR: Total Remission
TRAF6: tumor necrosis factor receptor-associated factor 6
Tregs: regulatory T cells
TUNEL: transferase-mediated dUTP Nick-End Labeling
USP14: ubiquitin-specific protease 14
WT-1: Wilms tumor 1
12-LOX: 12 - lipoxygenase
24h-UPE: 24-h urine protein excretion
